# Handwriting-based Alzheimer’s disease classification on the DARWIN dataset: Repeated nested validation, task-level reduction, and cross-validated explainability

**DOI:** 10.1371/journal.pone.0361117

**Published:** 2026-10-26

**Authors:** Tehreem Fatima, Tahira Anwar Lashari, Momina Moetesum, Sana Anwar Lashari

**Affiliations:** 1 Department of Computing, School of Electrical Engineering and Computer Science, National University of Sciences and Technology (NUST), Islamabad, Pakistan; 2 School of Nursing, Shandong Xiehe University, Jinan, Shandong, China; Polytechnic Institute of Leiria: Instituto Politecnico de Leiria, PORTUGAL

## Abstract

Online handwriting provides a low-burden source of cognitive–motor measurements, but small datasets can yield optimistic results when preprocessing, task selection, or explanation is not separated from evaluation. We evaluated eight classifiers on the DARWIN dataset (174 participants; 25 tasks; 450 predictors) using ten repetitions of 5-outer/3-inner-fold nested stratified cross-validation. All preprocessing, hyperparameter selection, and calibration were restricted to outer training data. We added a training-derived operating point, fully nested seven-task selection, cross-model subset transfer, foldwise SHAP analysis, and an all-feature SHAP sensitivity analysis. Random Forest had the highest descriptive ROC-AUC (0.938 ± 0.033 across 50 held-out folds). The Friedman omnibus test indicated model differences (p < 0.001), but no one of 28 paired comparisons remained significant after Holm correction. Training-derived thresholds (median 0.461) yielded held-out sensitivity 0.869 and specificity 0.831; these are exploratory operating characteristics, not a clinical cutoff. Nested seven-task selection achieved AUC 0.916 ± 0.037, compared with 0.956 ± 0.030 for all 25 tasks, and selected subsets were unstable (mean Jaccard 0.195). Cross-validated SHAP emphasized temporal variables, although an all-450-feature sensitivity analysis showed that individual ranks depend partly on ANOVA preselection. The study contributes an integrated, leakage-controlled evaluation rather than a new classifier or a validated diagnostic instrument; external prospective validation remains necessary.

## 1. Introduction

Alzheimer’s disease (AD) is a progressive neurodegenerative disorder associated with decline in memory, reasoning, and everyday functioning and is a leading cause of dementia worldwide [1]. Biomarker-supported diagnosis can involve imaging or cerebrospinal fluid procedures that are resource intensive and not suited to population-scale preliminary assessment [2]. Accessible behavioural measurements may therefore complement, but cannot replace, clinical evaluation.

Handwriting integrates attention, visuospatial processing, language, motor planning, and fine motor control. AD and related cognitive impairment have been associated with changes in writing speed, pausing, pressure, and stroke organisation [3,4]. Digitising tablets make these temporal and kinematic processes measurable, creating a possible non-invasive input for retrospective classification research.

The DARWIN benchmark contains online handwriting measurements from 174 participants (89 with AD and 85 healthy controls) who performed 25 tasks [5]. Prior studies have evaluated conventional classifiers, ensembles, feature-selection methods, deep models, and explainability approaches, but their reported performance is difficult to compare because data splits, tuning, task scope, and metrics differ.

Three methodological questions remain particularly important on this small cohort. First, apparent model rankings may change with the outer split and require repeated, paired evaluation. Second, a task subset chosen and scored on the same participants can overstate reduction performance. Third, feature explanations can become outcome-informed when ANOVA selection is performed once on the full labelled dataset. Threshold-free discrimination also does not by itself describe the sensitivity–specificity trade-off relevant to screening.

The novelty of this work is methodological and integrative, not algorithmic. Nested cross-validation, Random Forest, XGBoost, grouped greedy selection, and SHAP are established methods. The contribution is their auditable combination in one leakage-controlled analysis that (i) compares eight classifiers over 50 held-out folds with multiplicity-adjusted paired statistics; (ii) evaluates a complete task-selection procedure on participants excluded from selection; (iii) derives an operating threshold from training data only; and (iv) aligns foldwise SHAP values and tests their sensitivity to univariate preselection. These analyses are intended as a rigorous benchmark and hypothesis-generating assessment, not a clinically validated screening protocol.

## 2. Related work

Cilia et al. introduced DARWIN and reported an SVM-based benchmark [5]. Mitra and Rehman subsequently evaluated stacking under repeated validation [6], while Moreira et al. combined dimensionality reduction with an Explainable Boosting Machine [7]. Pannala et al. reported a voting ensemble with ANOVA selection [8]. These studies demonstrate the benchmark’s utility but do not provide head-to-head estimates under a common resampling design.

More recent DARWIN analyses include a hybrid transformer focused on a single task [9], stroke-level modelling [10], CNN explanation with DeepSHAP [11], and broader reviews of LIME and SHAP in AD modelling [12]. Other work has examined word semantics and phonology [13], PCA-based classification [14], sequential handwriting representations [15], and one-dimensional convolutional networks [16].

Handwriting assessment for neurological conditions has also been reviewed at the visual and procedural levels [17], and studies of Parkinson’s disease illustrate related feature and task-design considerations [18,19]. Generative augmentation [20], hybrid SHAP-SVM modelling [21], digital-biomarker reviews [22], and self-attention mechanisms [23] further show the diversity of available methods. Their validation protocols range from one train–test split to repeated or nested cross-validation, so numerical results should not be treated as a common leaderboard.

Accordingly, the present study does not claim a new classifier or feature-selection algorithm. It complements earlier work through a controlled repeated evaluation, honest task-selection assessment, training-only threshold derivation, and foldwise explanation workflow.

## 3. Materials and methods

### 3.1. Dataset and ethics

We analysed the public DARWIN data file [5] from the UCI Machine Learning Repository. After excluding the identifier and class columns, the analytic matrix contained 450 predictors: 18 engineered features for each of 25 tasks. The outcome was encoded as healthy control = 0 and AD = 1. The released file contained 85 controls and 89 participants with AD and no missing predictor values. It did not contain participant-level age, education, sex, or gender fields; therefore, demographic balance and covariate sensitivity analyses could not be performed.

Ethics and consent. This study is a secondary analysis of a publicly released, de-identified dataset. The original investigators reported institutional ethics approval and informed consent from participants or their legal representatives [5]. We recruited no participants, collected no new identifiable information, and performed no participant contact; no additional review was required for this secondary analysis.

### 3.2. Preprocessing and correlated predictors

For every inner and outer training split, a single pipeline fitted median imputation, an ANOVA F-test selector (SelectKBest; k ∈ {30, 60, 90}), and z-score scaling before fitting the classifier. The value of k and all model hyperparameters were chosen jointly by the inner cross-validation loop. The corresponding outer test fold was never used to estimate medians, F statistics, selected features, scaling parameters, calibration, or hyperparameters.

The 450 predictors are not independent. In particular, for every task and every participant, total_time equalled air_time plus paper_time exactly in the released file. This perfect linear relationship, together with other correlated kinematic measures, can divide tree and SHAP importance among substitutable predictors. We therefore interpret individual attribution ranks conditionally on the fitted pipeline and place greater weight on recurrent feature families than on one isolated rank.

### 3.3. Classifiers and hyperparameter search

We evaluated Logistic Regression [24] with C ∈ {0.5, 1, 2}; an RBF Support Vector Machine [25] with C ∈ {1, 5}, gamma = scale; KNN [26] with neighbours ∈ {3, 5} and weights ∈ {uniform, distance}; Gaussian Naïve Bayes without additional hyperparameters; Random Forest [27] with 300 trees and maximum depth ∈ {None, 12}; an MLP with hidden layers (64, 32), maximum 1,000 iterations, and alpha ∈ {10^−4^, 10^−3^}; and XGBoost [28] with 300 trees, maximum depth ∈ {4, 6}, learning rate 0.1, subsample 0.9, and column subsample 0.9. The stacking model [29] used the seven fixed base estimators, three-fold internal out-of-fold predictions, and Logistic Regression as the final estimator; its inner grid tuned ANOVA k. Implementations used scikit-learn [30] and the XGBoost Python API.

### 3.4. Repeated nested validation, thresholds, and statistics

Evaluation used stratified nested cross-validation [31]: five outer folds estimated performance and three inner folds selected hyperparameters by ROC-AUC [32]. The complete outer procedure was repeated with seeds 42–51, yielding 50 held-out evaluations per classifier. Within each outer training partition, the selected model was calibrated by three-fold isotonic calibration [33,34]. The conventional probability threshold of 0.50 was prespecified for the primary classification metrics. ROC-AUC remained the primary threshold-independent discrimination measure.

A secondary Random Forest operating point was learned separately in every outer fold. Three-fold out-of-fold probabilities from the outer training partition were used to select the threshold with maximum sensitivity among thresholds attaining at least 80% specificity; ties used the higher threshold. The selected threshold was then applied unchanged to the untouched outer test fold. This procedure estimates a screening-oriented trade-off without using test labels and does not establish a clinical cutoff. Sigmoid calibration is described by Platt [35], but was not used in the final reported pipeline.

We reported accuracy, macro-F1, sensitivity, specificity, Brier score [36], and expected calibration error in addition to ROC-AUC. For classifier comparison, each seed contributed one mean outer-fold AUC for every model. A Friedman omnibus test [37] was followed by all 28 paired two-sided Wilcoxon signed-rank comparisons with Holm family-wise correction [38]. The ten repetitions reuse the same participants and are not independent cohorts; tests and variability summaries are therefore interpreted conservatively. Training time included inner search, refitting, and calibration. Inference time was the median per-participant predict_proba time over repeated calls.

Computations used Python 3.12.13, NumPy 2.4.6, pandas 3.0.3, SciPy 1.18.0, scikit-learn 1.9.0, XGBoost 3.2.0, SHAP 0.52.0, and Matplotlib 3.10.8 on Linux with nine logical CPUs available (AMD EPYC 9V74) and 15.9 GiB RAM. No GPU was used.

### 3.5. Task-level greedy forward selection with swap refinement

The original analysis was labelled “SFFS-style,” but its procedure does not implement classical Sequential Forward Floating Selection, which interleaves conditional exclusions with inclusions [39]. We therefore renamed it task-level greedy forward selection with swap refinement (GFS-SR) and make no algorithmic novelty claim. All 18 predictors for a task entered or left together.

For an exact task budget k, GFS-SR began with an empty set and added the task giving the highest inner three-fold AUC until k tasks were present. It then evaluated every one-for-one exchange between a selected and unselected task, accepted the best strictly improving swap, and repeated until no swap improved AUC. Candidate subsets were cached; deterministic task-number order resolved equal scores. The search classifier was a lightweight Random Forest with 100 trees and maximum depth 5.

The same-cohort searches for k = 1–10 are retained only to document how the original seven-task hypothesis arose. For honest evaluation conditional on the originally proposed seven-task burden, the full GFS-SR search was rerun within each of five outer training folds using three-fold inner scores. The seven tasks selected without the outer participants were fitted on the outer training data and evaluated on the untouched fold. A full 25-task Random Forest with the same fixed search-model configuration was evaluated on the same outer splits. Task-selection frequency and pairwise Jaccard overlap quantified stability.

Finally, the fixed same-cohort seven-task set (Tasks 2, 5, 9, 10, 19, 21, and 24) was transferred without reselecting tasks to Logistic Regression, SVM-RBF, Random Forest, and XGBoost in five-fold nested model evaluation. Because that set was chosen using the full cohort, the transfer analysis is exploratory and tests model dependence; it is not an unbiased validation of the fixed subset.

### 3.6. Cross-validated SHAP analysis

SHAP [40] explanations were generated for Random Forest and XGBoost. In each of five outer folds, imputation, ANOVA k selection, scaling, tree hyperparameter selection, and fitting used only outer training data; SHAP values were calculated only for held-out participants. Selected k was 60 or 90. Absolute SHAP values were aligned by feature name, with zero contribution assigned when a feature was not selected in a fold, and then aggregated across all held-out participants. SHAP was applied to the tuned tree estimator before the separate calibration wrapper so that TreeExplainer described the fitted tree decision function.

We reported feature-, task-, and feature-type-level normalized mean absolute importance and contrasted correct with misclassified Random Forest predictions. To assess whether ANOVA could exclude interaction-relevant predictors, we repeated the five-fold held-out SHAP workflow with all 450 predictors and compared the combined rank profile and top-20 overlap. These explanation analyses describe model associations, not causal mechanisms or validated biomarkers.

## 4. Results

### 4.1. Repeated classifier comparison and operating point

Table 1 reports performance across 50 held-out outer folds. Random Forest had the highest observed AUC (0.938 ± 0.033; 2.5th–97.5th fold percentiles 0.868–0.992). At threshold 0.50 its accuracy was 0.856 ± 0.048, sensitivity 0.866 ± 0.069, and specificity 0.847 ± 0.095. Stacking had the highest observed accuracy (0.870) and macro-F1 (0.869). These are descriptive ranks.

**Table 1:** Repeated nested cross-validation performance at threshold 0.50. Values are mean ± SD across 50 held-out outer folds; the bracketed interval in the ROC-AUC column is the 2.5th–97.5th percentile envelope, not a confidence interval.

Model	ROC-AUC	Accuracy	Macro-F1	Sensitivity	Specificity
Random Forest	0.938 ± 0.033 [0.868, 0.992]	0.856 ± 0.048	0.855 ± 0.049	0.866 ± 0.069	0.847 ± 0.095
Stacking	0.930 ± 0.043 [0.840, 0.993]	0.870 ± 0.048	0.869 ± 0.048	0.885 ± 0.065	0.854 ± 0.082
XGBoost	0.923 ± 0.039 [0.839, 0.985]	0.841 ± 0.057	0.840 ± 0.058	0.853 ± 0.061	0.828 ± 0.098
Gaussian Naïve Bayes	0.918 ± 0.041 [0.839, 0.993]	0.865 ± 0.050	0.864 ± 0.051	0.886 ± 0.060	0.844 ± 0.088
SVM-RBF	0.913 ± 0.045 [0.837, 0.993]	0.846 ± 0.050	0.845 ± 0.050	0.859 ± 0.078	0.833 ± 0.083
MLP	0.910 ± 0.045 [0.843, 0.991]	0.852 ± 0.046	0.852 ± 0.046	0.855 ± 0.070	0.849 ± 0.083
Logistic Regression	0.902 ± 0.041 [0.845, 0.971]	0.827 ± 0.059	0.826 ± 0.059	0.822 ± 0.096	0.833 ± 0.086
KNN	0.890 ± 0.047 [0.786, 0.963]	0.828 ± 0.047	0.827 ± 0.047	0.805 ± 0.091	0.853 ± 0.082

The Friedman test indicated an overall difference among the eight classifiers (χ^2^(7) = 45.70, p < 0.001). Random Forest had the lowest mean rank (1.70), but none of the 28 paired Wilcoxon comparisons remained significant after Holm correction (minimum adjusted p = 0.055; S1 Table). Table 2 summarises rank, calibration, and computation. On the stated CPU, median outer-fold training time ranged from 0.115 s to 10.08 s; median inference was below 1.4 ms per participant for every model.

**Table 2:** Calibration, mean AUC rank, and computational timing. Brier and ECE are mean ± SD across 50 held-out folds. Timing is median [IQR] per outer fold.

Model	Mean rank	Brier	ECE	Training (s)	Inference (ms/person)
Random Forest	1.70	0.103 ± 0.027	0.123 ± 0.031	1.877 [1.856, 1.919]	0.927 [0.905, 0.961]
Stacking	2.35	0.104 ± 0.034	0.122 ± 0.032	10.080 [9.921, 10.376]	1.324 [1.293, 1.351]
XGBoost	3.70	0.115 ± 0.027	0.129 ± 0.032	0.526 [0.488, 0.547]	0.156 [0.154, 0.159]
Gaussian Naïve Bayes	4.10	0.108 ± 0.040	0.111 ± 0.037	0.115 [0.110, 0.124]	0.138 [0.136, 0.142]
SVM-RBF	4.80	0.116 ± 0.036	0.135 ± 0.036	0.177 [0.168, 0.189]	0.142 [0.140, 0.145]
MLP	5.45	0.116 ± 0.031	0.130 ± 0.034	0.577 [0.536, 0.630]	0.131 [0.129, 0.135]
Logistic Regression	6.20	0.129 ± 0.032	0.142 ± 0.038	0.273 [0.265, 0.288]	0.125 [0.123, 0.128]
KNN	7.70	0.131 ± 0.032	0.139 ± 0.041	0.285 [0.270, 0.313]	0.146 [0.145, 0.150]

The repeated held-out Random Forest ROC curves, accumulated threshold-0.50 confusion matrix, and pooled calibration plot are shown in Figs 1–3. Each participant contributed one held-out prediction per repetition to Figs 2 and 3; the counts are prediction instances rather than unique participants.

**Fig 1:**
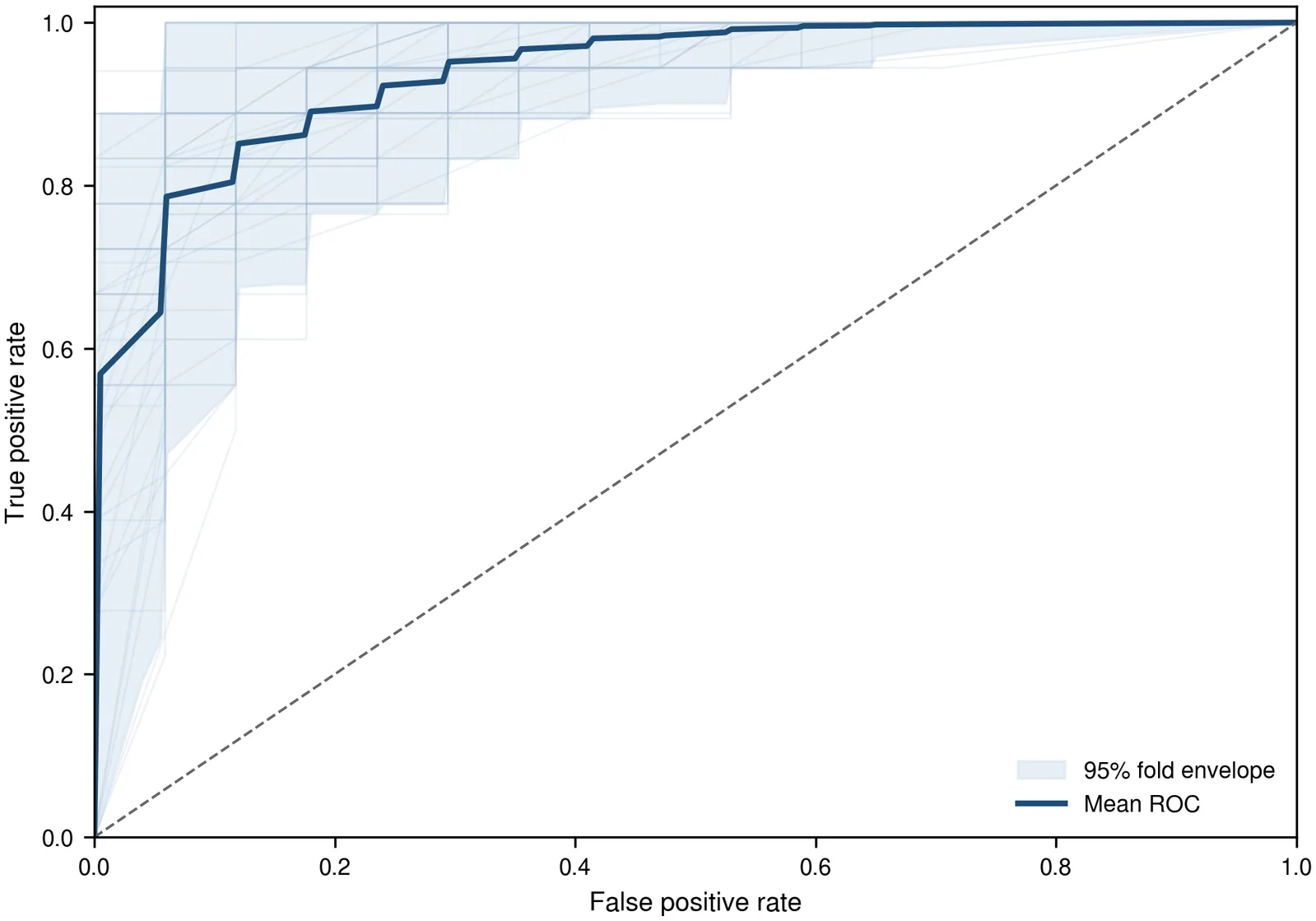
Random Forest ROC curves across 50 held-out outer folds. The dark line is the mean interpolated ROC; the shaded region is the 2.5th–97.5th percentile fold envelope. Thin lines show individual folds.

**Fig 2:**
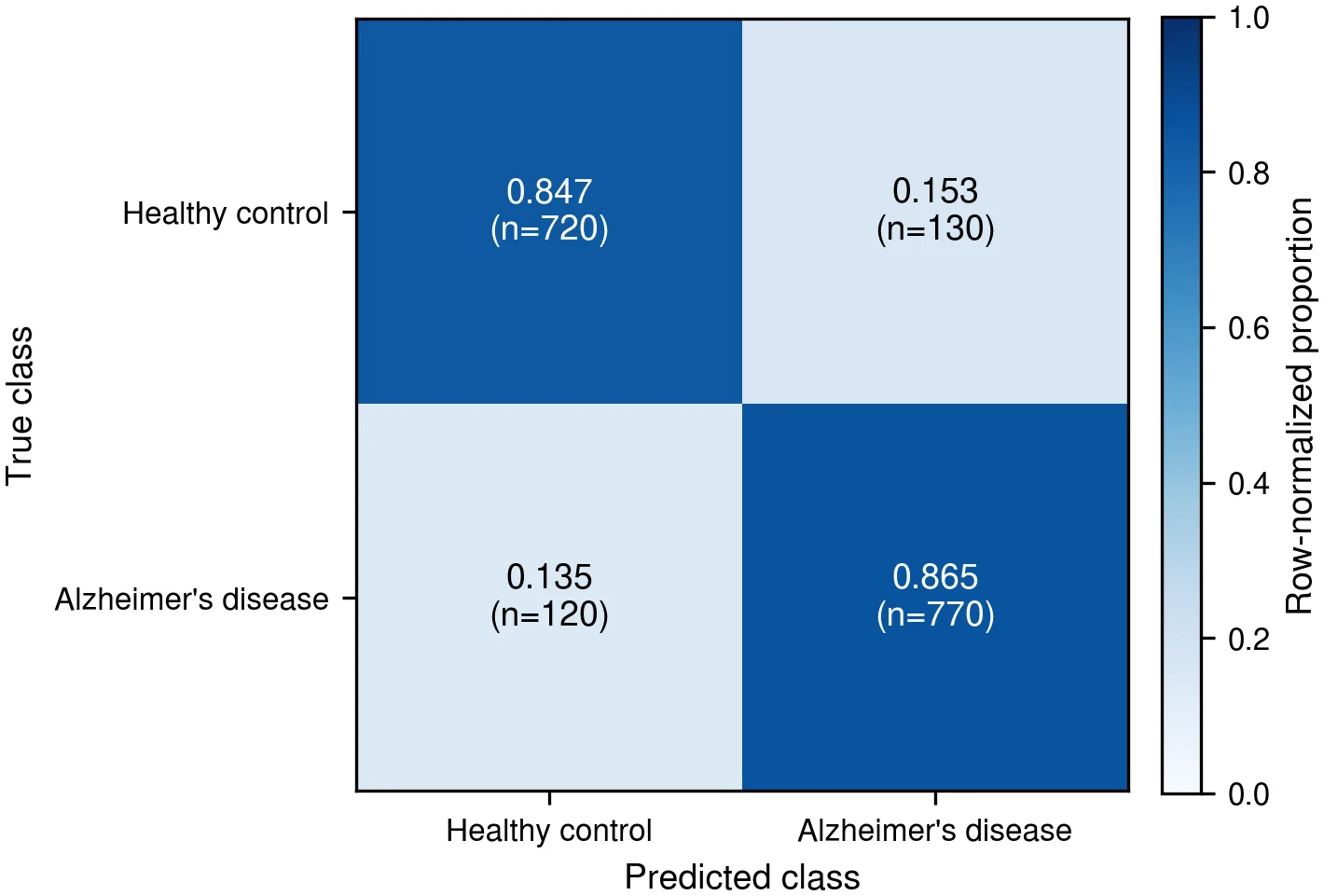
Accumulated Random Forest confusion matrix at threshold 0.50 across ten repeated five-fold evaluations. Rows are normalised; counts are held-out prediction instances.

**Fig 3:**
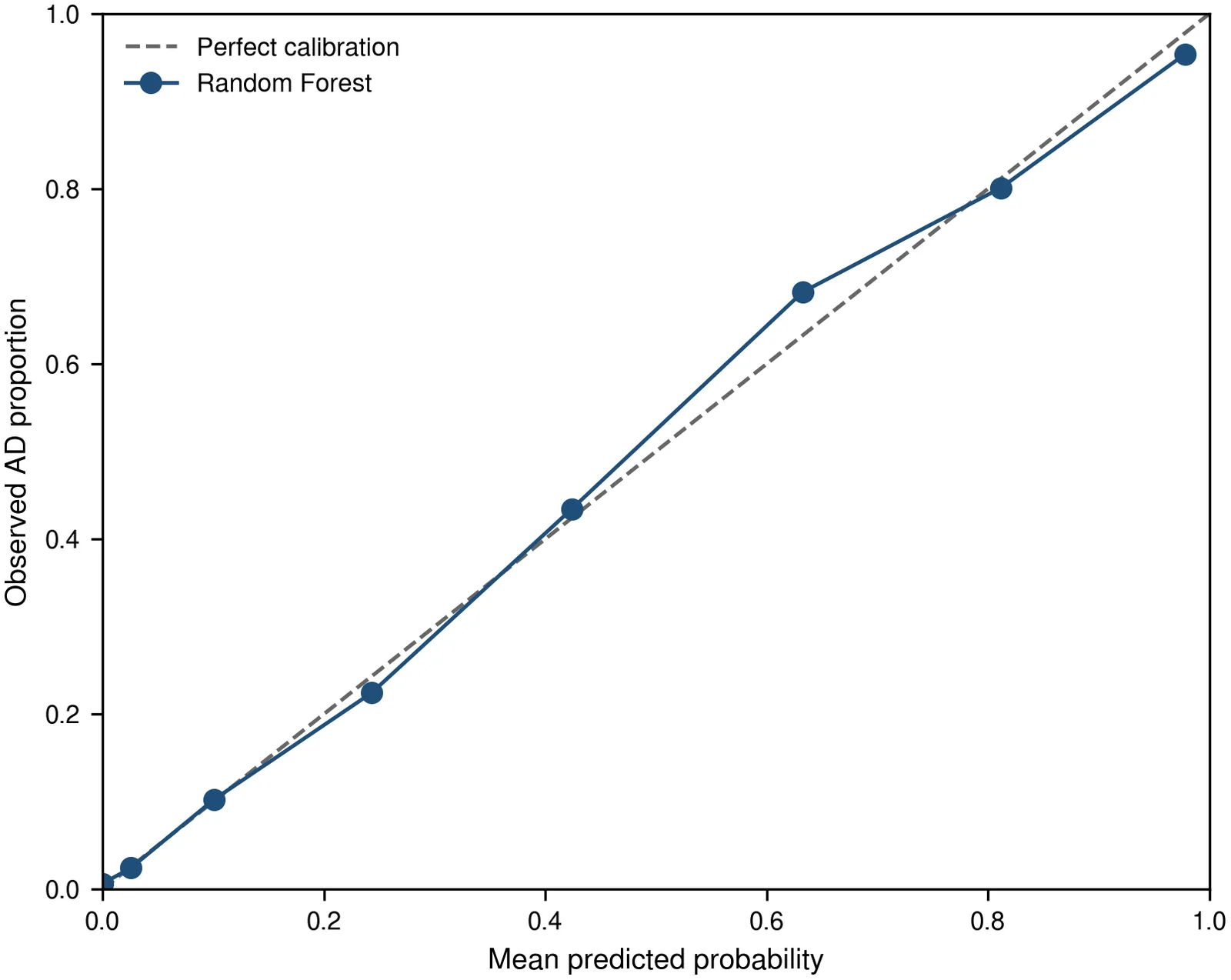
Random Forest calibration plot from repeated held-out predictions. Points use quantile bins; the dashed line indicates perfect calibration.

For the secondary operating-point analysis, fold-specific training-derived thresholds had median 0.461 (IQR 0.406–0.522; range 0.322–0.667). Applied to outer test folds, mean sensitivity was 0.869 ± 0.070 and specificity was 0.831 ± 0.113. The variation across folds precludes presenting one retrospectively fitted threshold as a clinical cutoff.

### 4.2. Task-wise performance

Single-task discrimination varied across the 25 tasks (Table 3). Task 23 (telephone number under dictation) had the highest task-wise AUC (0.871 with Random Forest), followed by Task 17 (copy six words into boxes; AUC 0.864) and Task 19 (postal-order fields; AUC 0.853). Task 22 achieved AUC 0.830 and is therefore reported as moderate, not as one of the strongest tasks. These task-wise estimates are descriptive and were not used as independent evidence of a clinical battery.

**Table 3:** Best model for each handwriting task under the submitted task-wise nested cross-validation analysis. Values are mean ± SD across five outer folds where available.

Task	Description	Best model	ROC-AUC	Accuracy	Macro-F1
1	Signature drawing	Random Forest	0.744 ± 0.079	0.650	0.640
2	Join two points with a horizontal line four times	Logistic Regression	0.826 ± 0.036	0.747	0.743
3	Join two points with a vertical line four times	Logistic Regression	0.741 ± 0.067	0.678	0.677
4	Retrace a 6-cm circle four times	Random Forest	0.770 ± 0.078	0.661	0.659
5	Retrace a 3-cm circle four times	Stacking	0.787 ± 0.065	0.718	0.713
6	Copy the letters “l”, “m”, and “p”	Logistic Regression	0.795 ± 0.016	0.707	0.702
7	Copy letters on adjacent rows	Stacking	0.832 ± 0.095	0.730	0.729
8	Write four lowercase “l” letters cursively	Logistic Regression	0.795 ± 0.050	0.718	0.716
9	Write four cursive “le” bigrams continuously	Random Forest	0.844 ± 0.051	0.753	0.749
10	Copy the word “sheet”	Random Forest	0.800 ± 0.102	0.695	0.684
11	Copy “sheet” above a line	XGBoost	0.770 ± 0.084	0.701	0.697
12	Copy the word “mum”	SVM-RBF	0.742 ± 0.064	0.684	0.683
13	Copy “mum” above a line	MLP	0.775 ± 0.037	0.712	0.711
14	Memorize and rewrite three words	XGBoost	0.832 ± 0.077	0.771	0.769
15	Copy “bottle” in reverse	Logistic Regression	0.810 ± 0.062	0.724	0.723
16	Copy “house” in reverse	SVM-RBF	0.798 ± 0.044	0.736	0.734
17	Copy six words into boxes	Random Forest	0.864 ± 0.055	0.793	0.792
18	Name and write a pictured object	MLP	0.785 ± 0.068	0.690	0.689
19	Copy the fields of a postal order	Random Forest	0.853 ± 0.054	0.742	0.734
20	Write a sentence under dictation	Random Forest	0.824 ± 0.061	0.707	0.701
21	Retrace a complex form	XGBoost	0.811 ± 0.078	0.724	0.723
22	Copy a telephone number	Random Forest	0.830 ± 0.049	0.747	0.744
23	Write a telephone number under dictation	Random Forest	0.871 ± 0.046	0.816	0.816
24	Draw a clock showing 11:05	XGBoost	0.785 ± 0.102	0.702	0.699
25	Copy a paragraph	Logistic Regression	0.828 ± 0.047	0.753	0.750

### 4.3. Task reduction and stability

The same-cohort GFS-SR search produced the previously reported maximum AUC of 0.9804 for Tasks 2, 5, 9, 10, 19, 21, and 24, compared with a same-cohort full-battery score of 0.9617. However, the fully nested, fixed-seven-task procedure achieved held-out AUC 0.916 ± 0.037, while the full 25-task comparator achieved 0.956 ± 0.030 on the same outer folds (Table 4). Thus, the unbiased comparison did not reproduce the apparent advantage of task reduction.

**Table 4:** Exploratory same-cohort GFS-SR scores and held-out fully nested task-selection results. Same-cohort search scores and outer-fold performance answer different questions and must not be compared as if they had the same validation design.

Configuration	Task set	Tasks	ROC-AUC	Interpretation
Same-cohort GFS-SR, k = 5	2,5,9,10,19	5	0.9769	Exploratory search score
Same-cohort GFS-SR, k = 7	2,5,9,10,19,21,24	7	0.9804	Exploratory search score
Same-cohort GFS-SR, k = 10	2,5,8,9,10,11,12,19,22,24	10	0.9802	Exploratory search score
Fully nested GFS-SR	Selected independently in each outer training fold	7/fold	0.916 ± 0.037	Held-out estimate
Fully nested full battery	Tasks 1–25	25	0.956 ± 0.030	Held-out comparator

Task selection was unstable across outer folds (mean pairwise Jaccard 0.195). Only Task 19 was selected in all five folds; Tasks 9 and 23 appeared in three folds each. The original Tasks 10 and 24 did not appear in any nested subset. Fig 4 shows the optimistic same-cohort search curve alongside nested selection frequency.

**Fig 4:**
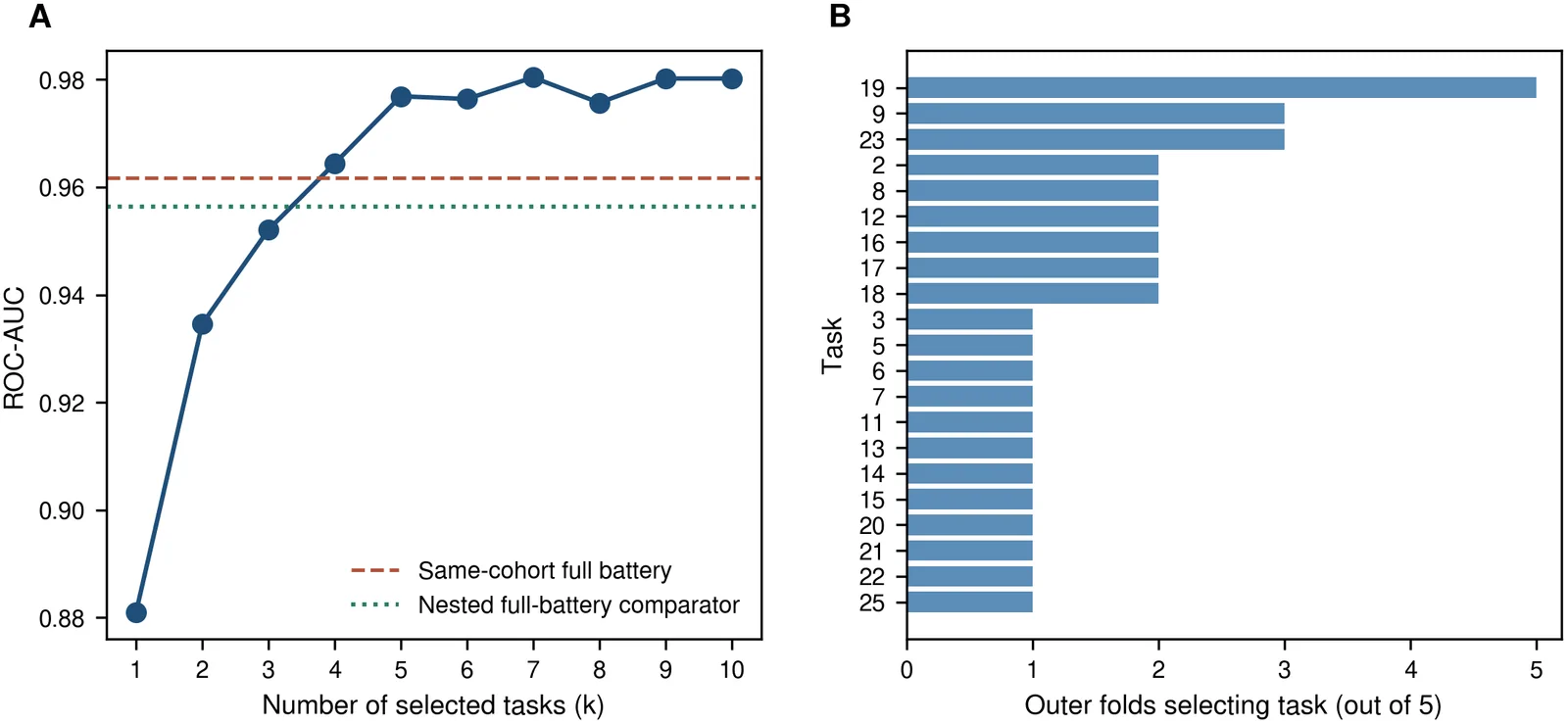
Task reduction results. (A) Same-cohort GFS-SR AUC by exact task budget; dashed and dotted lines show the same-cohort and nested full-battery comparators, respectively. (B) Frequency with which each task was selected inside five outer training folds.

The fixed same-cohort seven-task subset retained signal across all four tested classifier families (Table 5), but this transfer result cannot establish stability because the subset was chosen with the full labels. The nested task-selection result and low Jaccard overlap provide the appropriate evidence: the exact subset is not yet a validated replacement for the full battery.

**Table 5:** Exploratory transfer of the fixed same-cohort seven-task subset versus all 25 tasks. Values are mean ± SD over five outer folds. Task selection was not nested for the fixed subset, so these values must not be interpreted as an unbiased subset validation.

Model	Configuration	ROC-AUC	Accuracy	Sensitivity	Specificity
Logistic Regression	Fixed seven tasks	0.922 ± 0.053	0.862	0.865	0.859
Logistic Regression	All 25 tasks	0.923 ± 0.055	0.839	0.843	0.835
Random Forest	Fixed seven tasks	0.955 ± 0.041	0.897	0.910	0.882
Random Forest	All 25 tasks	0.948 ± 0.036	0.862	0.877	0.847
SVM-RBF	Fixed seven tasks	0.911 ± 0.048	0.868	0.898	0.835
SVM-RBF	All 25 tasks	0.904 ± 0.064	0.845	0.865	0.824
XGBoost	Fixed seven tasks	0.946 ± 0.037	0.856	0.933	0.776
XGBoost	All 25 tasks	0.916 ± 0.074	0.856	0.866	0.847

### 4.4. Cross-validated SHAP explanations

Fold-aligned held-out explanations emphasised temporal measurements. Total time, air time, and paper time accounted for combined normalised importance 0.216, 0.196, and 0.181, respectively. The leading combined features were total_time15, air_time6, paper_time17, air_time16, total_time9. Random Forest and XGBoost feature rankings are shown in Figs 5 and 6; task and feature-type aggregations are shown in Figs 7 and 8. Tasks 23, 17, and 15 had the highest task-level combined importance.

**Fig 5:**
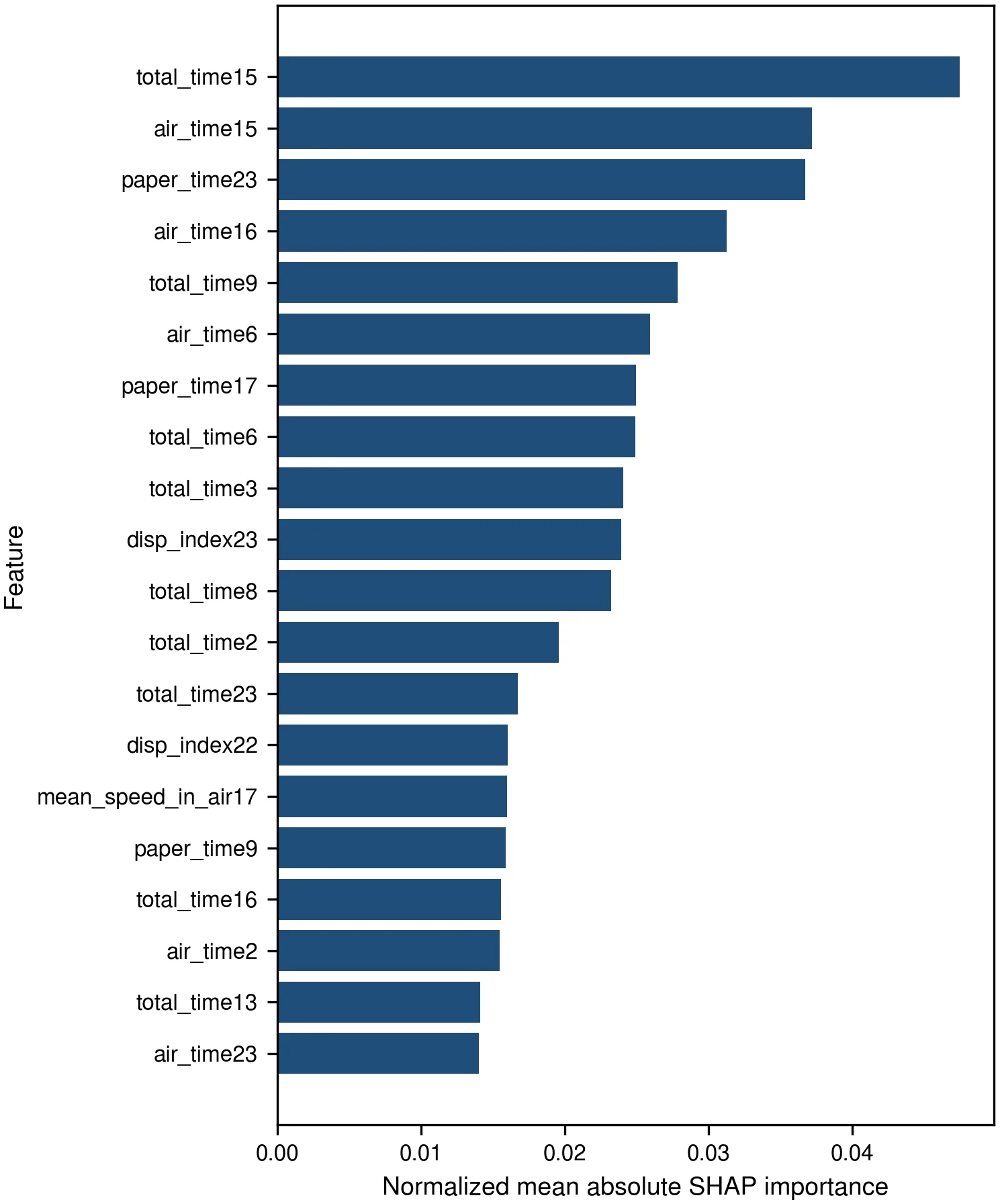
Random Forest fold-aggregated held-out SHAP ranking. Bars show normalised mean absolute SHAP importance for the 20 leading features.

**Fig 6:**
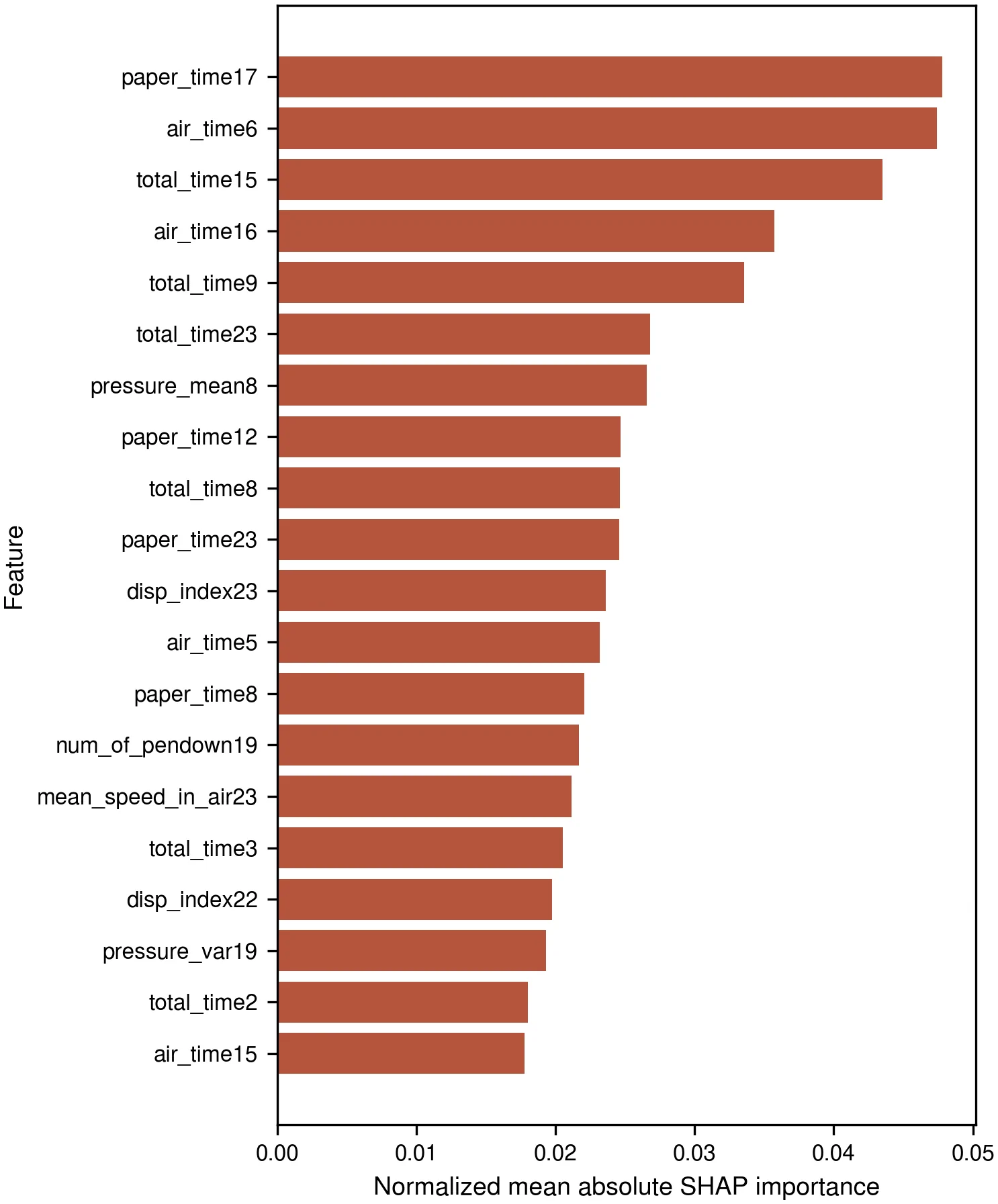
XGBoost fold-aggregated held-out SHAP ranking. Bars show normalised mean absolute SHAP importance for the 20 leading features.

**Fig 7:**
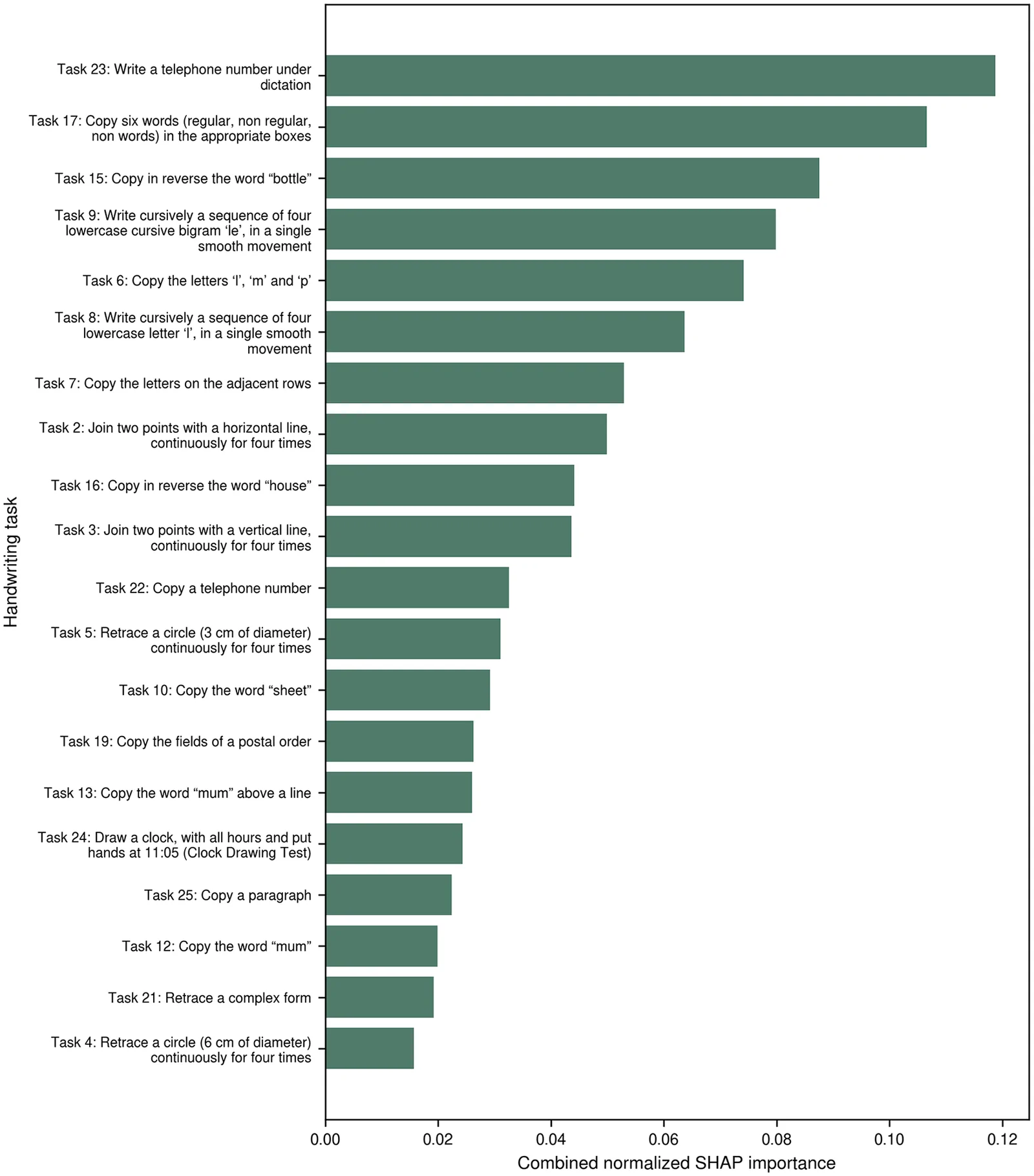
Task-level SHAP importance. Feature importance was summed within task and averaged across the normalised Random Forest and XGBoost profiles.

**Fig 8:**
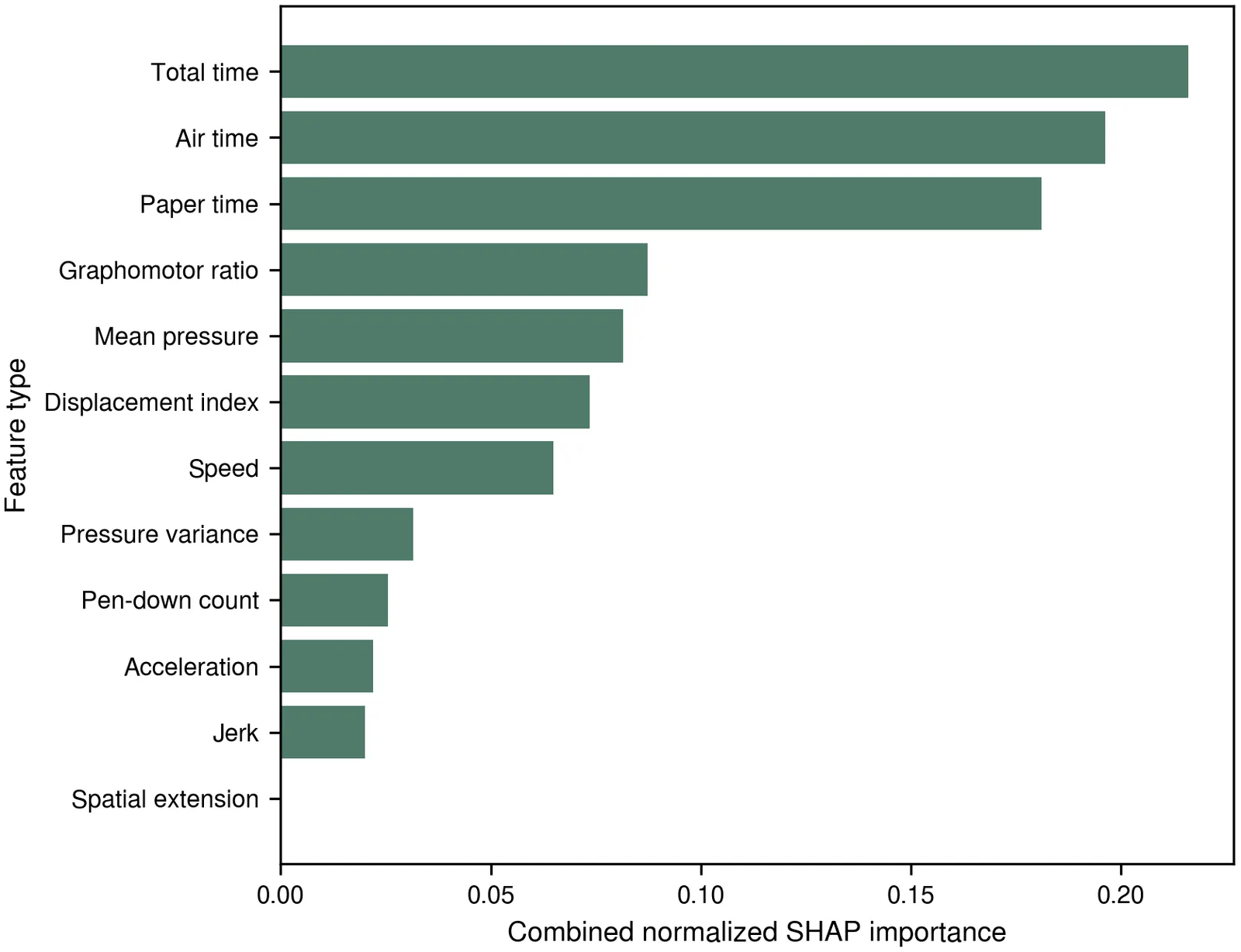
Feature-type SHAP importance. Values aggregate combined normalised importance across all tasks.

Table 6 lists the ten leading combined features with deliberately cautious exploratory interpretations. These interpretations do not convert SHAP associations into clinical biomarkers or disease mechanisms.

**Table 6:** Leading fold-aggregated SHAP features. Interpretations are exploratory associations and are not validated clinical biomarkers.

Rank	Feature	Task	Importance	Exploratory interpretation
1	total_time15	15	0.0455	Overall duration during reverse copying; associated with the fitted class but not evidence of executive dysfunction.
2	air_time6	6	0.0367	Pen-up duration during letter copying; may capture pauses or planning demands.
3	paper_time17	17	0.0364	Pen-contact duration during boxed word copying; combines visual, linguistic, and motor demands.
4	air_time16	16	0.0335	Pen-up duration during reverse word copying; associated with between-stroke hesitation.
5	total_time9	9	0.0307	Overall duration during continuous cursive bigram writing; associated with execution fluency.
6	paper_time23	23	0.0307	Active writing duration during dictated number production.
7	air_time15	15	0.0275	Pen-up duration during reverse word copying; an explanatory association only.
8	total_time8	8	0.0239	Overall duration during repeated cursive letter writing.
9	disp_index23	23	0.0238	Spatial displacement during dictated number writing.
10	total_time3	3	0.0223	Overall duration during repeated vertical-line production.

ANOVA preselection did affect individual rankings. When all 450 features were retained, the combined importance profile had Spearman ρ = 0.605 and 12/20 top features overlapped with the primary foldwise-selected analysis. Temporal variables remained prominent in both profiles, but features such as num_of_pendown19 and pressure_mean5 entered the all-feature top ranks. This sensitivity analysis confirms that feature selection can remove potentially relevant variables and that one exact SHAP ordering should not be overinterpreted.

### 4.5. Contrastive misclassification analysis

The five-fold Random Forest SHAP analysis classified 149 of 174 held-out participants correctly and misclassified 25. Misclassified cases had greater mean absolute contributions for paper_time10, pressure_var4, pressure_var5, num_of_pendown19, whereas correct cases had greater contributions for total_time15, air_time15, total_time2, total_time6. Fig 9 shows both the group profiles and their difference. This post hoc contrast describes uncertainty in this cohort and is not a participant-level error model.

**Fig 9:**
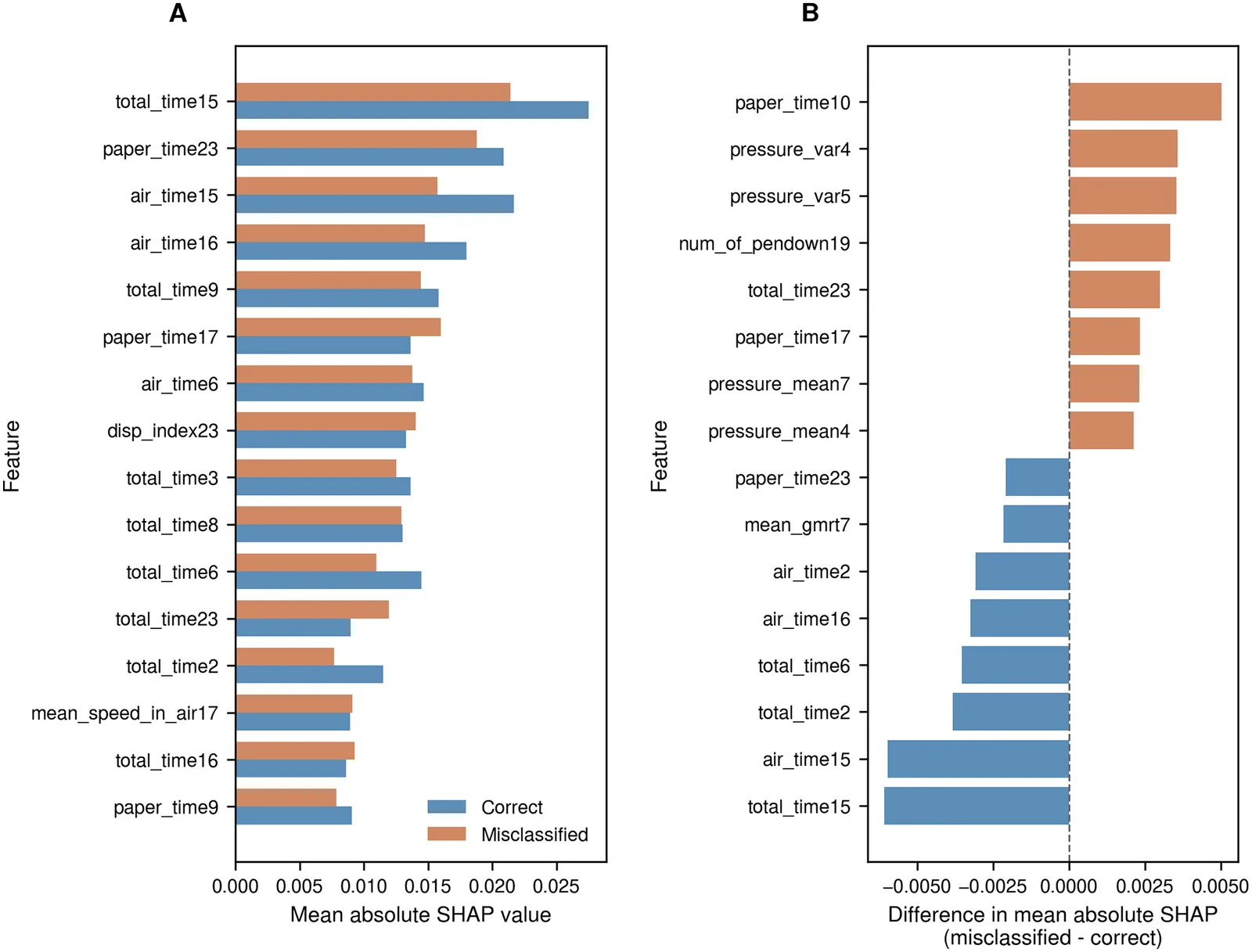
Contrastive Random Forest SHAP analysis. (A) Mean absolute contributions among correct and misclassified held-out predictions. (B) Misclassified-minus-correct difference for features with the largest absolute contrast. Positive values indicate larger contribution among misclassified cases.

### 4.6. Contextual comparison with prior DARWIN studies

Table 7 adds an explicit evaluation-protocol column to contextualise prior DARWIN results. The protocols differ in task scope, splitting, model selection, and metric calculation; their numerical values are not head-to-head estimates. The lower accuracy in the present analysis should therefore not be read as inferiority, just as higher single-split or same-cohort values should not be read as superiority.

**Table 7:** Contextual comparison with prior DARWIN studies. Values are reported as published and should be interpreted cautiously because validation protocols differ.

Study	Best reported model	Evaluation protocol	Reported performance
Cilia et al. [5]	SVM-based multi-classifier	Twenty random train–test evaluations; 5-fold tuning	Accuracy 94.28%; sensitivity 100%; specificity 88.24%
Mitra and Rehman [6]	Stacking	Repeated 10-fold and Monte Carlo CV	Accuracy 97.14%; sensitivity 95%; specificity 100%
Vimaladevi and Thangamani [14]	Random Forest	80/20 train–test split	Accuracy 94.29%
Moreira et al. [7]	Explainable Boosting Machine	Multiple data partitions	Accuracy 89 ± 5%
Pannala et al. [8]	Voting ensemble	Study-specific evaluation	Accuracy 100%
Gong et al. [9]	Hybrid transformer	Held-out test with 4-fold CV; Task 8	Accuracy 90.91%; F1 90.32%
Loodaricheh et al. [20]	XGBoost + VQ-VAE	5-fold CV	ROC-AUC 0.9586 ± 0.0046
This study	Random Forest	Repeated nested 10 × 5 outer folds; 3-fold inner tuning	Accuracy 0.856 ± 0.048; ROC-AUC 0.938 ± 0.033; sensitivity 0.866 ± 0.069; specificity 0.847 ± 0.095

## 5. Discussion

Repeated nested validation supports three conclusions. First, handwriting variables in DARWIN carry reproducible information about the recorded AD-versus-control labels. Random Forest had the highest observed AUC, while Stacking had the highest observed accuracy and macro-F1. The significant omnibus test indicates that model performance was not exchangeable overall, but no individual pair survived correction across 28 comparisons. We therefore avoid claims that one classifier is statistically superior to every alternative.

Second, the contrast between same-cohort and nested task-selection results demonstrates the magnitude and direction of selection bias. The original search maximum suggested that seven tasks outperformed the full battery, whereas the outer-fold procedure found lower mean AUC for selected seven-task sets and substantial subset instability. The fixed subset’s cross-model transfer is useful for generating future protocol hypotheses but cannot undo its whole-cohort selection. Prospective testing should predefine the task set and evaluate it once in a new cohort.

Third, temporal variables were recurrent across foldwise SHAP models and the all-feature sensitivity analysis. This pattern is compatible with prior reports that temporal efficiency and stroke organisation differ with cognitive impairment [3,41]. However, SHAP values are associations within fitted models. They cannot show that AD caused slowing, hesitation, or altered planning, and correlated variables can share or exchange importance.

Demographic confounding remains unresolved. Normal ageing itself affects handwriting speed and fluency [42]. Because DARWIN’s released analytic file contains no age or education variables, we could neither compare groups nor adjust the models. Statements about temporal features are therefore limited to association with class membership in this dataset. Future releases and external cohorts should include appropriately governed demographic variables and report stratified or adjusted analyses.

The operating-point analysis illustrates another limitation of retrospective screening claims. Although thresholds were derived without outer-test labels, they varied materially across folds and did not always attain 80% specificity in new participants. A clinical threshold must be prespecified and validated at the intended prevalence, site, device, and use case, with consequences of false negatives and false positives evaluated prospectively.

Other limitations include the small, single-dataset sample; absence of an external cohort; repeated reuse of the same participants; binary AD-versus-control labels rather than mild cognitive impairment, differential diagnosis, or stage; device-specific engineered predictors; perfect and near-perfect multicollinearity; and explanations conditional on model and preprocessing choices. The present results support reproducible retrospective methodology, not autonomous diagnosis or a clinically validated instrument.

## 6. Conclusions

Across 50 held-out outer folds, Random Forest achieved mean ROC-AUC 0.938, but multiplicity-adjusted pairwise tests did not establish universal superiority over the other classifiers. The training-only operating point quantified a sensitivity–specificity trade-off without defining a clinical threshold.

Fully nested seven-task selection achieved mean AUC 0.916 compared with 0.956 for all 25 tasks, and the selected subsets were unstable. The original seven-task set should therefore remain a hypothesis for external prospective testing.

Foldwise SHAP analyses consistently gave substantial importance to temporal handwriting variables, while an all-feature sensitivity analysis showed that exact ranks depend on preselection. The main contribution is an integrated, leakage-controlled and auditable evaluation framework; clinical translation requires independent multi-site validation with demographic covariates and prespecified tasks and thresholds.

## Supporting information

S1 Table All 28 paired Wilcoxon signed-rank comparisons of repetition-level mean AUCs with Holm correction. (DOCX)

S1 Code Reproducible analysis code, data audit, fold-level outputs, and publication-figure generation workflow. (ZIP)
