# Supplementary material for: Handwriting-based Alzheimer’s disease classification on the DARWIN dataset: Repeated nested validation, task-level reduction, and cross-validated explainability
Source: PLoS One. 2026 Oct 26;21(10):e0361117. doi: 10.1371/journal.pone.0361117 (PMC13704832; doi:10.1371/journal.pone.0361117)
Supplement: Supplementary file 1 [file pone.0361117.s001.docx]

**S1 Table. Complete non-parametric classifier comparisons**

Supporting information for manuscript PONE-D-26-28296

Each of ten split seeds contributed one mean outer-fold ROC-AUC per classifier. The Friedman omnibus test used these paired repetition-level values. All 28 two-sided Wilcoxon signed-rank comparisons were adjusted together by the Holm procedure. Repeated splits reuse the same 174 participants; inferential results are interpreted conservatively.

**Friedman χ²(7) = 45.696, p < 0.001.**

Table 1. Mean AUC ranks across ten paired repetitions; lower rank is better.

| **Model** | **Mean rank** |
| --- | --- |
| Random Forest | 1.70 |
| Stacking | 2.35 |
| XGBoost | 3.70 |
| Gaussian Naïve Bayes | 4.10 |
| SVM-RBF | 4.80 |
| MLP | 5.45 |
| Logistic Regression | 6.20 |
| KNN | 7.70 |

Table 2. All paired post hoc comparisons with Holm family-wise correction.

| **Model A** | **Model B** | **Mean AUC A** | **Mean AUC B** | **Mean Δ** | **Median Δ** | **W** | **Raw p** | **Holm p** | **Significant** |
| --- | --- | --- | --- | --- | --- | --- | --- | --- | --- |
| KNN | Gaussian Naïve Bayes | 0.8898 | 0.9184 | -0.0285 | -0.0305 | 0.0 | 0.002 | 0.055 | No |
| KNN | Stacking | 0.8898 | 0.9298 | -0.0399 | -0.0394 | 0.0 | 0.002 | 0.055 | No |
| KNN | XGBoost | 0.8898 | 0.9225 | -0.0327 | -0.0332 | 0.0 | 0.002 | 0.055 | No |
| Logistic Regression | Random Forest | 0.9021 | 0.9382 | -0.0361 | -0.0351 | 0.0 | 0.002 | 0.055 | No |
| Logistic Regression | Stacking | 0.9021 | 0.9298 | -0.0277 | -0.0282 | 0.0 | 0.002 | 0.055 | No |
| Random Forest | KNN | 0.9382 | 0.8898 | +0.0483 | +0.0456 | 0.0 | 0.002 | 0.055 | No |
| Random Forest | MLP | 0.9382 | 0.9102 | +0.0280 | +0.0276 | 0.0 | 0.002 | 0.055 | No |
| Logistic Regression | XGBoost | 0.9021 | 0.9225 | -0.0204 | -0.0188 | 1.0 | 0.004 | 0.082 | No |
| MLP | Stacking | 0.9102 | 0.9298 | -0.0196 | -0.0185 | 0.0 | 0.004 | 0.082 | No |
| SVM-RBF | Random Forest | 0.9128 | 0.9382 | -0.0253 | -0.0188 | 1.0 | 0.004 | 0.082 | No |
| SVM-RBF | Stacking | 0.9128 | 0.9298 | -0.0169 | -0.0153 | 2.0 | 0.006 | 0.105 | No |
| Gaussian Naïve Bayes | Stacking | 0.9184 | 0.9298 | -0.0114 | -0.0087 | 3.0 | 0.010 | 0.166 | No |
| KNN | MLP | 0.8898 | 0.9102 | -0.0203 | -0.0238 | 3.0 | 0.010 | 0.166 | No |
| Logistic Regression | KNN | 0.9021 | 0.8898 | +0.0123 | +0.0090 | 3.0 | 0.010 | 0.166 | No |
| Random Forest | Gaussian Naïve Bayes | 0.9382 | 0.9184 | +0.0198 | +0.0244 | 3.0 | 0.010 | 0.166 | No |
| Random Forest | XGBoost | 0.9382 | 0.9225 | +0.0157 | +0.0177 | 3.0 | 0.010 | 0.166 | No |
| MLP | XGBoost | 0.9102 | 0.9225 | -0.0123 | -0.0079 | 4.0 | 0.014 | 0.166 | No |
| SVM-RBF | KNN | 0.9128 | 0.8898 | +0.0230 | +0.0220 | 4.0 | 0.014 | 0.166 | No |
| Logistic Regression | Gaussian Naïve Bayes | 0.9021 | 0.9184 | -0.0163 | -0.0202 | 6.0 | 0.027 | 0.273 | No |
| Logistic Regression | SVM-RBF | 0.9021 | 0.9128 | -0.0107 | -0.0095 | 7.0 | 0.037 | 0.334 | No |
| Random Forest | Stacking | 0.9382 | 0.9298 | +0.0084 | +0.0059 | 10.0 | 0.084 | 0.672 | No |
| XGBoost | Stacking | 0.9225 | 0.9298 | -0.0073 | -0.0074 | 11.0 | 0.105 | 0.738 | No |
| Logistic Regression | MLP | 0.9021 | 0.9102 | -0.0081 | -0.0142 | 15.0 | 0.232 | 1.000 | No |
| Gaussian Naïve Bayes | MLP | 0.9184 | 0.9102 | +0.0082 | +0.0070 | 16.0 | 0.275 | 1.000 | No |
| SVM-RBF | XGBoost | 0.9128 | 0.9225 | -0.0097 | -0.0025 | 16.0 | 0.275 | 1.000 | No |
| SVM-RBF | Gaussian Naïve Bayes | 0.9128 | 0.9184 | -0.0055 | -0.0091 | 18.0 | 0.375 | 1.000 | No |
| Gaussian Naïve Bayes | XGBoost | 0.9184 | 0.9225 | -0.0041 | -0.0046 | 20.0 | 0.492 | 1.000 | No |
| SVM-RBF | MLP | 0.9128 | 0.9102 | +0.0027 | +0.0061 | 20.0 | 0.492 | 1.000 | No |
