# Supplementary material for: Handwriting-based Alzheimer’s disease classification on the DARWIN dataset: Repeated nested validation, task-level reduction, and cross-validated explainability
Source: PLoS One. 2026 Oct 26;21(10):e0361117. doi: 10.1371/journal.pone.0361117 (PMC13704832; doi:10.1371/journal.pone.0361117)
Supplement: Supplementary file 2 [file pone.0361117.s004.docx]

**Response to Reviewers**

PONE-D-26-28296 | PLOS ONE | Revision dated 23 July 2026

| **Original title** | Handwriting-Based Alzheimer’s Disease Detection: A Comparative Machine Learning Framework with Task-Optimised Screening and Explainability Analysis on the DARWIN Dataset |
| --- | --- |
| **Revised title** | Handwriting-based Alzheimer’s disease classification on the DARWIN dataset: repeated nested validation, task-level reduction, and cross-validated explainability |
| **First author** | Tehreem Fatima |
| **Files supplied** | Clean manuscript; marked manuscript; response letter; S1 statistical table; S1 Code; Fig1–Fig9 TIFF files |

# Dear Dr. Shokouhifar and Reviewers,

Thank you for the detailed and constructive evaluation. We have substantially revised the manuscript and rerun the analyses needed to resolve the methodological concerns. The revision is deliberately more conservative: it no longer claims a new algorithm, a statistically superior classifier, or a validated seven-task clinical protocol.

The principal changes are: ten repeated nested evaluations for all eight classifiers; a Friedman test and all 28 Holm-adjusted paired comparisons; training-only operating-point derivation; outer-nested seven-task selection with stability analysis; cross-model subset transfer labelled exploratory; foldwise SHAP with an all-450-feature sensitivity analysis; explicit demographic and multicollinearity limitations; corrected GFS-SR terminology; complete hyperparameters and timing; and PLOS formatting/data/ethics/citation corrections.

Below, each comment is reproduced and answered. Locations refer to the clean revised manuscript. The marked copy uses blue text with yellow highlighting for substantive revisions; the response below also records what was removed or reinterpreted.

# Detailed table of corrections

| **ID** | **Reviewer/editor request** | **Correction and evidence** | **Location** |
| --- | --- | --- | --- |
| JR1 | Ensure that the manuscript meets PLOS ONE style requirements, including file naming. | Completed. We prepared a clean unlocked DOCX and a separate marked copy. The manuscript uses a single-column, 12-point Times New Roman layout, double-spaced prose, continuous line numbering, page numbers, no more than three heading levels, Vancouver-style numbered references, and PLOS-compatible file names. Figures are not embedded; each caption is placed immediately after the paragraph that first cites the figure. Separate 300-dpi RGB LZW TIFF files are supplied as Fig1.tif–Fig9.tif. | Entire manuscript; separate figure files |
| JR2 | Add the DOI/accession number or a direct link for every dataset in the Data Availability Statement. | Completed. The revised statement gives both the direct UCI DARWIN page and the persistent dataset DOI https://doi.org/10.24432/C55D0K. It also identifies S1 Code as the source of the complete revised code, fold-level predictions, result tables, and figure workflow. | Data availability |
| JR3 | The ethics statement should appear only in the Methods section. | Completed. The ethics and consent statement now appears only in Section 3.1. The separate post-manuscript Institutional Review Board and Informed Consent sections were removed. The remaining statement clearly identifies this work as secondary analysis of public de-identified data and cites the original investigators’ approval and consent report. | Methods, Section 3.1 |
| JR4 | Refer to Figures 1–8 in the manuscript text. | Completed. Figs 1–3 are cited in Section 4.1, Fig 4 in Section 4.3, and Figs 5–8 in Section 4.4. The revised contrastive analysis is also cited as Fig 9 in Section 4.5. Every figure has a unique caption immediately after its first-citing paragraph. | Results, Sections 4.1, 4.3–4.5 |
| JR5 | Refer to Tables 3 and 4 in the manuscript text. | Completed. Table 3 is explicitly cited in the task-wise results and Table 4 is explicitly cited in the nested task-reduction results. We also checked and cited Tables 1, 2, and 5–7 at first mention. | Results, Sections 4.2 and 4.3 |
| JR6 | Evaluate reviewer-recommended references for relevance. | Completed. We evaluated the requested literature and added Pudil, Novovičová and Kittler (1994) to distinguish classical SFFS from our non-classical search. We also added two directly relevant sources on age, education, handwriting performance, and cognitive impairment. The citations are used to support a limitation, not to imply that unavailable demographic data were analysed. | Methods 3.5; Discussion; Refs 39, 41, 42 |
| R1.1 | Given that DARWIN, Random Forest, XGBoost, SFFS and SHAP already exist, where exactly is the main innovation? Is it algorithmic or the combination and clinical framework? | We agree that the submitted version overstated algorithmic novelty. The revision now states explicitly that the contribution is methodological and integrative, not a new classifier, selection algorithm, or clinical instrument. The specific addition is one auditable framework that combines: repeated leakage-controlled nested comparison over 50 held-out folds; all-model paired non-parametric statistics; training-only threshold derivation; outer-nested grouped task selection; foldwise SHAP; and an all-450-feature explanation sensitivity check. We changed the title, abstract, Introduction, and Conclusion to remove the implication of algorithmic invention or a validated clinical framework. | Title; Abstract; Introduction; Conclusion |
| R1.2 | What does the paper add that previous DARWIN studies of models, ensembles, feature selection and explainability did not? | The revision distinguishes our contribution by validation question, not by claiming a higher score. Prior reports use heterogeneous single-split, repeated, task-specific, or study-specific protocols. We added a protocol column to Table 7 and caution against direct numerical ranking. Our added evidence is: split-sensitivity across ten outer seeds; fully nested evaluation of the task-selection procedure itself; a training-derived operating point evaluated on untouched folds; cross-model subset transfer labelled exploratory; foldwise out-of-sample explanations; and sensitivity of those explanations to ANOVA removal. These analyses expose optimism and instability that a single best score does not show. | Introduction; Related work; Table 7; Discussion |
| R1.3 | Is comparison of eight models under joint nested cross-validation an independent scientific innovation or a standard reproducible baseline? | We now call it a controlled, reproducible baseline. Nested cross-validation is an established requirement, not an independent algorithmic innovation. Its role here is to create a common experimental control across eight classifiers and to support the additional repeated, task-level, threshold, and explanation analyses. Ranking language has been changed from “best/superior” to “highest observed” unless supported by corrected tests. | Introduction; Methods 3.4; Results 4.1; Discussion |
| R1.3.1 | Since SFFS is well known, what is innovative about using it? Was it merely applied to DARWIN or changed? | We agree that there is no SFFS algorithmic innovation. More importantly, the submitted procedure was not classical SFFS: it used greedy forward growth followed by one-for-one swaps at fixed k. We renamed it “task-level greedy forward selection with swap refinement” (GFS-SR), cited the classical Pudil et al. method, and added exact steps, termination, caching, and tie-breaking. The grouped use of all 18 predictors per task is an application design choice, not a new feature-selection algorithm. | Methods 3.5; Results 4.3; Fig 4; Ref 39 |
| R1.4 | Are imputation, ANOVA selection, standardisation and calibration all completed inside each fold of nested cross-validation? | Yes, and the revised Methods now make the nesting explicit. Within every outer training partition, the inner pipeline refits median imputation, jointly tunes ANOVA k ∈ {30, 60, 90} with model hyperparameters, refits scaling, and then fits three-fold isotonic calibration. No outer-test values inform any of these steps. The threshold is additionally learned from out-of-fold probabilities inside the outer training partition. The SHAP workflow independently refits imputation, ANOVA, scaling, tuning, and the tree model in each outer training fold. | Methods 3.2, 3.4, 3.6 |
| R1.5 | Could the higher seven-task AUC be overfitting because task selection and reporting used the same 174 people? | Yes. We agree and now demonstrate it rather than only mentioning it. The original same-cohort score of 0.9804 is retained solely as an exploratory search score. When the seven-task search was performed inside each outer training fold, mean held-out AUC fell to 0.916 ± 0.037, compared with 0.956 ± 0.030 for all 25 tasks. The apparent same-cohort advantage therefore did not generalise. | Methods 3.5; Results 4.3; Table 4; Discussion |
| R1.6 | Should task selection be performed inside a separate outer nested validation before proposing a seven-task screening protocol? | Implemented. We fixed the seven-task burden corresponding to the original hypothesis, but selected the identities of the seven tasks using only each outer training partition and three-fold inner scores. Only then was the selected set evaluated on the untouched fold. The full 25-task comparator used the same outer splits and search-model configuration. Because the nested result was lower and unstable, we no longer call the fixed subset a clinical screening protocol. | Methods 3.5; Results 4.3; Table 4; Fig 4 |
| R1.7 | Are the selected tasks stable for SVM, XGBoost or Logistic Regression, or optimised only for the lightweight Random Forest? | The selection objective is Random-Forest-specific, and we now say so plainly. Across five outer Random Forest searches, mean pairwise Jaccard overlap was only 0.195; only Task 19 appeared in all folds. We also transferred the fixed same-cohort subset to Logistic Regression, SVM-RBF, Random Forest and XGBoost (Table 5). It retained predictive signal, but those values remain optimistic because the fixed subset was chosen with all labels. We therefore do not claim universal task stability or model-independent optimality. | Methods 3.5; Results 4.3; Table 5; Fig 4 |
| R1.8 | For screening, false negatives are costly. Why use ROC-AUC rather than sensitivity at a fixed specificity? | ROC-AUC remains primary because it is threshold-independent and supports fair model comparison before a clinical operating point has been validated. We added the requested screening-oriented analysis: within each outer training set, the threshold maximising sensitivity while meeting at least 80% specificity was selected and applied unchanged to the outer test set. Mean held-out sensitivity was 0.869 and specificity 0.831. Because specificity can shift in unseen data, we report the achieved test specificity rather than implying it was guaranteed. | Methods 3.4; Results 4.1; Discussion |
| R1.9 | What is the final classification threshold? | The prespecified threshold for the primary classification metrics is 0.50. For the secondary screening-oriented analysis, the threshold was not fixed after looking at test data: one value was learned within each outer training partition. Across 50 folds, the median was 0.461 (IQR 0.406–0.522; range 0.322–0.667). We explicitly state that this distribution does not define a clinical cutoff. | Methods 3.4; Results 4.1 |
| R1.10 | Could clinically important features have been removed by ANOVA before SHAP? | Yes; univariate selection can exclude variables whose contribution is conditional or interaction-based. We addressed both leakage and sensitivity. First, ANOVA selection is now refitted separately in each outer training fold and SHAP is computed only for held-out participants. Second, we repeated the entire held-out SHAP analysis with all 450 predictors. The all-feature and primary combined profiles had Spearman ρ = 0.605 and 12/20 top-feature overlap. Temporal variables remained prominent, but individual ranks changed; we now state this explicitly and provide the sensitivity outputs. | Methods 3.6; Results 4.4; Discussion; S1 Code |
| R2.1 | What is the configured value for the final classification threshold? | The primary configured threshold is 0.50. We added this value to the Methods, Table 1 caption, Fig 2 caption, and Results. We also added a separate training-derived threshold analysis targeting at least 80% specificity; its median threshold was 0.461, but it is not presented as a fixed clinical cutoff. | Methods 3.4; Results 4.1; Table 1; Fig 2 |
| R2.2 | The principal idea and innovation are not adequately conveyed. | We rewrote the title, abstract, final Introduction paragraph, and Conclusion. The principal idea is an integrated audit of model, task, threshold, and explanation reliability on a small benchmark. The novel contribution is the coordinated validation design and the evidence it reveals—not a new Random Forest, XGBoost, SHAP, or selection algorithm. We also removed “clinically feasible protocol” language unsupported by external validation. | Title; Abstract; Introduction; Conclusion |
| R2.3 | What differs between this paper and ideas already reviewed in the literature? | The Related Work now separates methods from validation questions, and Table 7 provides a protocol column. Relative to previous DARWIN reports, this revision supplies repeated paired nested estimates for all eight classifiers, complete multiplicity-adjusted statistics, fully nested task selection, training-only threshold derivation, foldwise out-of-sample SHAP, and an all-feature explanation sensitivity analysis. We do not compare published scores as if they came from one benchmark split. | Related work; Methods; Table 7 |
| R2.4 | Was SFFS optimised? If not, explain the proposed development or extension. | The method was neither classical SFFS nor a new optimisation. We corrected the terminology to GFS-SR and now specify its exact-k forward phase, exhaustive one-for-one swap phase, strict improvement criterion, termination rule, caching, and deterministic tie-breaking. The only domain adaptation is grouped selection of whole tasks (18 features per task). We make no claim that this is an extension of Pudil et al.; the scientific analysis concerns its nested evaluation. | Methods 3.5; Ref 39 |
| R3.1 | Provide a comprehensive non-parametric statistical comparison among all classifiers. | Implemented. Each of ten split seeds contributes one paired mean outer-fold AUC per classifier. The Friedman omnibus test was significant (χ²(7) = 45.70, p < 0.001). We then performed all 28 paired two-sided Wilcoxon tests and corrected them together with Holm’s procedure. No pair remained significant (minimum adjusted p = 0.055). Mean ranks are in Table 2; all pairwise estimates, raw p values, and adjusted p values are in S1 Table. | Methods 3.4; Results 4.1; Table 2; S1 Table |
| R3.2 | Task selection and evaluation on the same dataset may cause optimistic bias; discuss explicitly and recommend external validation. | We agree. The revision now gives an empirical nested estimate and changes the interpretation. The same-cohort AUC 0.9804 is labelled a search score, not performance evidence. The fully nested seven-task procedure achieved 0.916 versus 0.956 for the full battery, and task overlap was low (Jaccard 0.195). External prospective validation of a prespecified subset is now stated as necessary. | Results 4.3; Discussion; Conclusion |
| R3.3 | Add an evaluation-protocol column to the prior-study table and caution against direct numerical comparisons. | Completed. Table 7 now lists the reported model, evaluation protocol, and published metric for each study. The surrounding text explicitly states that split design, task scope, model selection, and metric calculation differ, so the table is contextual rather than a leaderboard. | Results 4.6; Table 7 |
| R3.4 | Provide detailed hyperparameter search spaces and optimisation procedures for every model. | Completed in Section 3.3. We list every grid: Logistic Regression C; SVM C/gamma/kernel; KNN neighbours/weights; Random Forest trees/depth; MLP architecture/alpha/iterations; XGBoost trees/depth plus fixed learning and sampling settings; no additional GaussianNB grid; and the Stacking composition and internal CV. Section 3.2 clarifies that ANOVA k ∈ {30, 60, 90} is jointly tuned in the inner loop. | Methods 3.2–3.4 |
| R3.5 | Report computational resources, training time, and inference time. | Completed. We report Python/package versions, CPU, available logical CPUs, RAM, operating system, and absence of GPU use. Table 2 gives median [IQR] outer-fold training and per-participant inference time for every classifier. Median training ranged from 0.115 s (GaussianNB) to 10.08 s (Stacking); median inference remained under 1.4 ms/person. | Methods 3.4; Results 4.1; Table 2 |
| R4.M1 | Repeat the single nested CV over multiple random seeds and report the outer-fold AUC distribution. | Implemented with ten deterministic seeds (42–51), producing 50 held-out outer-fold evaluations for each of eight classifiers. Random Forest mean AUC was 0.938 ± 0.033; its 2.5th–97.5th fold percentiles were 0.868–0.992. Table 1 reports every model’s mean, SD and percentile envelope, and Fig 1 shows all 50 held-out ROC curves. | Methods 3.4; Results 4.1; Table 1; Fig 1 |
| R4.M2 | Refit SHAP-stage feature selection within a cross-validated wrapper or state the whole-sample leakage limitation. | Implemented. For each of five outer folds, the imputer, ANOVA selector, k, scaler, tree hyperparameters, and model are fitted using only the outer training participants; explanations are computed only for the outer test participants and aligned by feature name. We additionally ran the all-450-feature held-out sensitivity analysis. Figures 5–9 and Table 6 were regenerated from these foldwise outputs, replacing the whole-cohort explanation figures. | Methods 3.6; Results 4.4–4.5; Figs 5–9 |
| R4.M3 | Report/control age and education or soften claims because these demographic confound handwriting dynamics. | We agree with the concern. The released analytic file contains ID, 450 handwriting predictors, and class only; it has no age or education columns. We therefore did not invent, infer, or impute demographic attributes. The limitation is now explicit in Methods and Discussion; mechanistic wording has been replaced by “associated with class membership.” We added directly relevant references on normal ageing, education-adjusted cognitive assessment, and handwriting dynamics. | Methods 3.1; Discussion; Refs 41–42 |
| R4.M4 | Correct the imprecise SFFS terminology or provide precise pseudocode, termination and tie-breaking. | Corrected. The method is now GFS-SR, explicitly distinguished from classical SFFS. Section 3.5 specifies grouped task units, forward addition, exhaustive one-for-one swap refinement, strict-improvement termination, cache use, and deterministic task-number tie-breaking. Pudil et al. (1994) is cited for the classical method. | Methods 3.5; Ref 39 |
| R4.M5 | Clarify whether ANOVA k ∈ {30,60,90} is tuned inside the inner CV or selected post hoc. | Clarified and verified in code: k is a GridSearchCV pipeline hyperparameter jointly tuned with model parameters inside every three-fold inner loop. It is never selected from outer-test results. The fitted imputer, F scores, selected columns, scaler, model, and calibration remain confined to the outer training data. | Methods 3.2 and 3.4 |
| R4.m1 | Use a broader Friedman/post hoc comparison or temper rankings because several models cluster. | Both changes were made. We report the Friedman test, all 28 Holm-adjusted Wilcoxon comparisons, and mean ranks. Although Random Forest had the lowest observed rank, no pair remained significant after correction. The text therefore uses “highest observed AUC” and does not claim statistical superiority. | Results 4.1; Tables 1–2; S1 Table |
| R4.m2 | Acknowledge multicollinearity, including total_time = air_time + paper_time, as a caveat for SHAP stability. | Completed and quantified. A data audit confirmed the identity exactly for all 25 tasks and all 174 records. Section 3.2 explains that correlated variables can arbitrarily share or exchange tree/SHAP importance. Results and Discussion therefore emphasise recurrent feature families and the all-feature sensitivity analysis rather than one exact feature rank. | Methods 3.2; Results 4.4; Discussion; S1 Code data audit |
| R4.m3 | Task 22 AUC 0.830 should not be described as a high-ranking task. | Corrected. Section 4.2 now identifies Tasks 23, 17, and 19 as the three strongest task-wise AUCs and explicitly describes Task 22’s 0.830 value as moderate. Table 3 retains the exact result. | Results 4.2; Table 3 |
| R4.m4 | Relabel the speculative “Clinical Significance” column in the SHAP table. | Corrected. The column is now “Exploratory interpretation,” and the caption states that the entries are explanatory associations rather than validated biomarkers. Mechanistic claims such as executive planning difficulty were removed or reframed as task demands that cannot be inferred causally from SHAP. | Results 4.4; Table 6 |
| R4.m5 | Cite Pudil et al. (1994) and a source addressing demographic confounds in handwriting assessment. | Completed. Pudil et al. is now Reference 39 and is used to distinguish classical floating search from GFS-SR. References 41 and 42 address cognitive impairment, education-adjusted assessment, normal ageing, and handwriting performance. They support the new confounding limitation and softened interpretation. | Methods 3.5; Discussion; Refs 39, 41, 42 |

# Point-by-point responses

# Journal requirement

## JR1

*Comment: Ensure that the manuscript meets PLOS ONE style requirements, including file naming.*

**Response:** Completed. We prepared a clean unlocked DOCX and a separate marked copy. The manuscript uses a single-column, 12-point Times New Roman layout, double-spaced prose, continuous line numbering, page numbers, no more than three heading levels, Vancouver-style numbered references, and PLOS-compatible file names. Figures are not embedded; each caption is placed immediately after the paragraph that first cites the figure. Separate 300-dpi RGB LZW TIFF files are supplied as Fig1.tif–Fig9.tif.

**Manuscript location:** *Entire manuscript; separate figure files*

## JR2

*Comment: Add the DOI/accession number or a direct link for every dataset in the Data Availability Statement.*

**Response:** Completed. The revised statement gives both the direct UCI DARWIN page and the persistent dataset DOI https://doi.org/10.24432/C55D0K. It also identifies S1 Code as the source of the complete revised code, fold-level predictions, result tables, and figure workflow.

**Manuscript location:** *Data availability*

## JR3

*Comment: The ethics statement should appear only in the Methods section.*

**Response:** Completed. The ethics and consent statement now appears only in Section 3.1. The separate post-manuscript Institutional Review Board and Informed Consent sections were removed. The remaining statement clearly identifies this work as secondary analysis of public de-identified data and cites the original investigators’ approval and consent report.

**Manuscript location:** *Methods, Section 3.1*

## JR4

*Comment: Refer to Figures 1–8 in the manuscript text.*

**Response:** Completed. Figs 1–3 are cited in Section 4.1, Fig 4 in Section 4.3, and Figs 5–8 in Section 4.4. The revised contrastive analysis is also cited as Fig 9 in Section 4.5. Every figure has a unique caption immediately after its first-citing paragraph.

**Manuscript location:** *Results, Sections 4.1, 4.3–4.5*

## JR5

*Comment: Refer to Tables 3 and 4 in the manuscript text.*

**Response:** Completed. Table 3 is explicitly cited in the task-wise results and Table 4 is explicitly cited in the nested task-reduction results. We also checked and cited Tables 1, 2, and 5–7 at first mention.

**Manuscript location:** *Results, Sections 4.2 and 4.3*

## JR6

*Comment: Evaluate reviewer-recommended references for relevance.*

**Response:** Completed. We evaluated the requested literature and added Pudil, Novovičová and Kittler (1994) to distinguish classical SFFS from our non-classical search. We also added two directly relevant sources on age, education, handwriting performance, and cognitive impairment. The citations are used to support a limitation, not to imply that unavailable demographic data were analysed.

**Manuscript location:** *Methods 3.5; Discussion; Refs 39, 41, 42*

# Reviewer 1

## R1.1

*Comment: Given that DARWIN, Random Forest, XGBoost, SFFS and SHAP already exist, where exactly is the main innovation? Is it algorithmic or the combination and clinical framework?*

**Response:** We agree that the submitted version overstated algorithmic novelty. The revision now states explicitly that the contribution is methodological and integrative, not a new classifier, selection algorithm, or clinical instrument. The specific addition is one auditable framework that combines: repeated leakage-controlled nested comparison over 50 held-out folds; all-model paired non-parametric statistics; training-only threshold derivation; outer-nested grouped task selection; foldwise SHAP; and an all-450-feature explanation sensitivity check. We changed the title, abstract, Introduction, and Conclusion to remove the implication of algorithmic invention or a validated clinical framework.

**Manuscript location:** *Title; Abstract; Introduction; Conclusion*

## R1.2

*Comment: What does the paper add that previous DARWIN studies of models, ensembles, feature selection and explainability did not?*

**Response:** The revision distinguishes our contribution by validation question, not by claiming a higher score. Prior reports use heterogeneous single-split, repeated, task-specific, or study-specific protocols. We added a protocol column to Table 7 and caution against direct numerical ranking. Our added evidence is: split-sensitivity across ten outer seeds; fully nested evaluation of the task-selection procedure itself; a training-derived operating point evaluated on untouched folds; cross-model subset transfer labelled exploratory; foldwise out-of-sample explanations; and sensitivity of those explanations to ANOVA removal. These analyses expose optimism and instability that a single best score does not show.

**Manuscript location:** *Introduction; Related work; Table 7; Discussion*

## R1.3

*Comment: Is comparison of eight models under joint nested cross-validation an independent scientific innovation or a standard reproducible baseline?*

**Response:** We now call it a controlled, reproducible baseline. Nested cross-validation is an established requirement, not an independent algorithmic innovation. Its role here is to create a common experimental control across eight classifiers and to support the additional repeated, task-level, threshold, and explanation analyses. Ranking language has been changed from “best/superior” to “highest observed” unless supported by corrected tests.

**Manuscript location:** *Introduction; Methods 3.4; Results 4.1; Discussion*

## R1.3.1

*Comment: Since SFFS is well known, what is innovative about using it? Was it merely applied to DARWIN or changed?*

**Response:** We agree that there is no SFFS algorithmic innovation. More importantly, the submitted procedure was not classical SFFS: it used greedy forward growth followed by one-for-one swaps at fixed k. We renamed it “task-level greedy forward selection with swap refinement” (GFS-SR), cited the classical Pudil et al. method, and added exact steps, termination, caching, and tie-breaking. The grouped use of all 18 predictors per task is an application design choice, not a new feature-selection algorithm.

**Manuscript location:** *Methods 3.5; Results 4.3; Fig 4; Ref 39*

## R1.4

*Comment: Are imputation, ANOVA selection, standardisation and calibration all completed inside each fold of nested cross-validation?*

**Response:** Yes, and the revised Methods now make the nesting explicit. Within every outer training partition, the inner pipeline refits median imputation, jointly tunes ANOVA k ∈ {30, 60, 90} with model hyperparameters, refits scaling, and then fits three-fold isotonic calibration. No outer-test values inform any of these steps. The threshold is additionally learned from out-of-fold probabilities inside the outer training partition. The SHAP workflow independently refits imputation, ANOVA, scaling, tuning, and the tree model in each outer training fold.

**Manuscript location:** *Methods 3.2, 3.4, 3.6*

## R1.5

*Comment: Could the higher seven-task AUC be overfitting because task selection and reporting used the same 174 people?*

**Response:** Yes. We agree and now demonstrate it rather than only mentioning it. The original same-cohort score of 0.9804 is retained solely as an exploratory search score. When the seven-task search was performed inside each outer training fold, mean held-out AUC fell to 0.916 ± 0.037, compared with 0.956 ± 0.030 for all 25 tasks. The apparent same-cohort advantage therefore did not generalise.

**Manuscript location:** *Methods 3.5; Results 4.3; Table 4; Discussion*

## R1.6

*Comment: Should task selection be performed inside a separate outer nested validation before proposing a seven-task screening protocol?*

**Response:** Implemented. We fixed the seven-task burden corresponding to the original hypothesis, but selected the identities of the seven tasks using only each outer training partition and three-fold inner scores. Only then was the selected set evaluated on the untouched fold. The full 25-task comparator used the same outer splits and search-model configuration. Because the nested result was lower and unstable, we no longer call the fixed subset a clinical screening protocol.

**Manuscript location:** *Methods 3.5; Results 4.3; Table 4; Fig 4*

## R1.7

*Comment: Are the selected tasks stable for SVM, XGBoost or Logistic Regression, or optimised only for the lightweight Random Forest?*

**Response:** The selection objective is Random-Forest-specific, and we now say so plainly. Across five outer Random Forest searches, mean pairwise Jaccard overlap was only 0.195; only Task 19 appeared in all folds. We also transferred the fixed same-cohort subset to Logistic Regression, SVM-RBF, Random Forest and XGBoost (Table 5). It retained predictive signal, but those values remain optimistic because the fixed subset was chosen with all labels. We therefore do not claim universal task stability or model-independent optimality.

**Manuscript location:** *Methods 3.5; Results 4.3; Table 5; Fig 4*

## R1.8

*Comment: For screening, false negatives are costly. Why use ROC-AUC rather than sensitivity at a fixed specificity?*

**Response:** ROC-AUC remains primary because it is threshold-independent and supports fair model comparison before a clinical operating point has been validated. We added the requested screening-oriented analysis: within each outer training set, the threshold maximising sensitivity while meeting at least 80% specificity was selected and applied unchanged to the outer test set. Mean held-out sensitivity was 0.869 and specificity 0.831. Because specificity can shift in unseen data, we report the achieved test specificity rather than implying it was guaranteed.

**Manuscript location:** *Methods 3.4; Results 4.1; Discussion*

## R1.9

*Comment: What is the final classification threshold?*

**Response:** The prespecified threshold for the primary classification metrics is 0.50. For the secondary screening-oriented analysis, the threshold was not fixed after looking at test data: one value was learned within each outer training partition. Across 50 folds, the median was 0.461 (IQR 0.406–0.522; range 0.322–0.667). We explicitly state that this distribution does not define a clinical cutoff.

**Manuscript location:** *Methods 3.4; Results 4.1*

## R1.10

*Comment: Could clinically important features have been removed by ANOVA before SHAP?*

**Response:** Yes; univariate selection can exclude variables whose contribution is conditional or interaction-based. We addressed both leakage and sensitivity. First, ANOVA selection is now refitted separately in each outer training fold and SHAP is computed only for held-out participants. Second, we repeated the entire held-out SHAP analysis with all 450 predictors. The all-feature and primary combined profiles had Spearman ρ = 0.605 and 12/20 top-feature overlap. Temporal variables remained prominent, but individual ranks changed; we now state this explicitly and provide the sensitivity outputs.

**Manuscript location:** *Methods 3.6; Results 4.4; Discussion; S1 Code*

# Reviewer 2

## R2.1

*Comment: What is the configured value for the final classification threshold?*

**Response:** The primary configured threshold is 0.50. We added this value to the Methods, Table 1 caption, Fig 2 caption, and Results. We also added a separate training-derived threshold analysis targeting at least 80% specificity; its median threshold was 0.461, but it is not presented as a fixed clinical cutoff.

**Manuscript location:** *Methods 3.4; Results 4.1; Table 1; Fig 2*

## R2.2

*Comment: The principal idea and innovation are not adequately conveyed.*

**Response:** We rewrote the title, abstract, final Introduction paragraph, and Conclusion. The principal idea is an integrated audit of model, task, threshold, and explanation reliability on a small benchmark. The novel contribution is the coordinated validation design and the evidence it reveals—not a new Random Forest, XGBoost, SHAP, or selection algorithm. We also removed “clinically feasible protocol” language unsupported by external validation.

**Manuscript location:** *Title; Abstract; Introduction; Conclusion*

## R2.3

*Comment: What differs between this paper and ideas already reviewed in the literature?*

**Response:** The Related Work now separates methods from validation questions, and Table 7 provides a protocol column. Relative to previous DARWIN reports, this revision supplies repeated paired nested estimates for all eight classifiers, complete multiplicity-adjusted statistics, fully nested task selection, training-only threshold derivation, foldwise out-of-sample SHAP, and an all-feature explanation sensitivity analysis. We do not compare published scores as if they came from one benchmark split.

**Manuscript location:** *Related work; Methods; Table 7*

## R2.4

*Comment: Was SFFS optimised? If not, explain the proposed development or extension.*

**Response:** The method was neither classical SFFS nor a new optimisation. We corrected the terminology to GFS-SR and now specify its exact-k forward phase, exhaustive one-for-one swap phase, strict improvement criterion, termination rule, caching, and deterministic tie-breaking. The only domain adaptation is grouped selection of whole tasks (18 features per task). We make no claim that this is an extension of Pudil et al.; the scientific analysis concerns its nested evaluation.

**Manuscript location:** *Methods 3.5; Ref 39*

# Reviewer 3

## R3.1

*Comment: Provide a comprehensive non-parametric statistical comparison among all classifiers.*

**Response:** Implemented. Each of ten split seeds contributes one paired mean outer-fold AUC per classifier. The Friedman omnibus test was significant (χ²(7) = 45.70, p < 0.001). We then performed all 28 paired two-sided Wilcoxon tests and corrected them together with Holm’s procedure. No pair remained significant (minimum adjusted p = 0.055). Mean ranks are in Table 2; all pairwise estimates, raw p values, and adjusted p values are in S1 Table.

**Manuscript location:** *Methods 3.4; Results 4.1; Table 2; S1 Table*

## R3.2

*Comment: Task selection and evaluation on the same dataset may cause optimistic bias; discuss explicitly and recommend external validation.*

**Response:** We agree. The revision now gives an empirical nested estimate and changes the interpretation. The same-cohort AUC 0.9804 is labelled a search score, not performance evidence. The fully nested seven-task procedure achieved 0.916 versus 0.956 for the full battery, and task overlap was low (Jaccard 0.195). External prospective validation of a prespecified subset is now stated as necessary.

**Manuscript location:** *Results 4.3; Discussion; Conclusion*

## R3.3

*Comment: Add an evaluation-protocol column to the prior-study table and caution against direct numerical comparisons.*

**Response:** Completed. Table 7 now lists the reported model, evaluation protocol, and published metric for each study. The surrounding text explicitly states that split design, task scope, model selection, and metric calculation differ, so the table is contextual rather than a leaderboard.

**Manuscript location:** *Results 4.6; Table 7*

## R3.4

*Comment: Provide detailed hyperparameter search spaces and optimisation procedures for every model.*

**Response:** Completed in Section 3.3. We list every grid: Logistic Regression C; SVM C/gamma/kernel; KNN neighbours/weights; Random Forest trees/depth; MLP architecture/alpha/iterations; XGBoost trees/depth plus fixed learning and sampling settings; no additional GaussianNB grid; and the Stacking composition and internal CV. Section 3.2 clarifies that ANOVA k ∈ {30, 60, 90} is jointly tuned in the inner loop.

**Manuscript location:** *Methods 3.2–3.4*

## R3.5

*Comment: Report computational resources, training time, and inference time.*

**Response:** Completed. We report Python/package versions, CPU, available logical CPUs, RAM, operating system, and absence of GPU use. Table 2 gives median [IQR] outer-fold training and per-participant inference time for every classifier. Median training ranged from 0.115 s (GaussianNB) to 10.08 s (Stacking); median inference remained under 1.4 ms/person.

**Manuscript location:** *Methods 3.4; Results 4.1; Table 2*

# Reviewer 4

## R4.M1

*Comment: Repeat the single nested CV over multiple random seeds and report the outer-fold AUC distribution.*

**Response:** Implemented with ten deterministic seeds (42–51), producing 50 held-out outer-fold evaluations for each of eight classifiers. Random Forest mean AUC was 0.938 ± 0.033; its 2.5th–97.5th fold percentiles were 0.868–0.992. Table 1 reports every model’s mean, SD and percentile envelope, and Fig 1 shows all 50 held-out ROC curves.

**Manuscript location:** *Methods 3.4; Results 4.1; Table 1; Fig 1*

## R4.M2

*Comment: Refit SHAP-stage feature selection within a cross-validated wrapper or state the whole-sample leakage limitation.*

**Response:** Implemented. For each of five outer folds, the imputer, ANOVA selector, k, scaler, tree hyperparameters, and model are fitted using only the outer training participants; explanations are computed only for the outer test participants and aligned by feature name. We additionally ran the all-450-feature held-out sensitivity analysis. Figures 5–9 and Table 6 were regenerated from these foldwise outputs, replacing the whole-cohort explanation figures.

**Manuscript location:** *Methods 3.6; Results 4.4–4.5; Figs 5–9*

## R4.M3

*Comment: Report/control age and education or soften claims because these demographic confound handwriting dynamics.*

**Response:** We agree with the concern. The released analytic file contains ID, 450 handwriting predictors, and class only; it has no age or education columns. We therefore did not invent, infer, or impute demographic attributes. The limitation is now explicit in Methods and Discussion; mechanistic wording has been replaced by “associated with class membership.” We added directly relevant references on normal ageing, education-adjusted cognitive assessment, and handwriting dynamics.

**Manuscript location:** *Methods 3.1; Discussion; Refs 41–42*

## R4.M4

*Comment: Correct the imprecise SFFS terminology or provide precise pseudocode, termination and tie-breaking.*

**Response:** Corrected. The method is now GFS-SR, explicitly distinguished from classical SFFS. Section 3.5 specifies grouped task units, forward addition, exhaustive one-for-one swap refinement, strict-improvement termination, cache use, and deterministic task-number tie-breaking. Pudil et al. (1994) is cited for the classical method.

**Manuscript location:** *Methods 3.5; Ref 39*

## R4.M5

*Comment: Clarify whether ANOVA k ∈ {30,60,90} is tuned inside the inner CV or selected post hoc.*

**Response:** Clarified and verified in code: k is a GridSearchCV pipeline hyperparameter jointly tuned with model parameters inside every three-fold inner loop. It is never selected from outer-test results. The fitted imputer, F scores, selected columns, scaler, model, and calibration remain confined to the outer training data.

**Manuscript location:** *Methods 3.2 and 3.4*

## R4.m1

*Comment: Use a broader Friedman/post hoc comparison or temper rankings because several models cluster.*

**Response:** Both changes were made. We report the Friedman test, all 28 Holm-adjusted Wilcoxon comparisons, and mean ranks. Although Random Forest had the lowest observed rank, no pair remained significant after correction. The text therefore uses “highest observed AUC” and does not claim statistical superiority.

**Manuscript location:** *Results 4.1; Tables 1–2; S1 Table*

## R4.m2

*Comment: Acknowledge multicollinearity, including total_time = air_time + paper_time, as a caveat for SHAP stability.*

**Response:** Completed and quantified. A data audit confirmed the identity exactly for all 25 tasks and all 174 records. Section 3.2 explains that correlated variables can arbitrarily share or exchange tree/SHAP importance. Results and Discussion therefore emphasise recurrent feature families and the all-feature sensitivity analysis rather than one exact feature rank.

**Manuscript location:** *Methods 3.2; Results 4.4; Discussion; S1 Code data audit*

## R4.m3

*Comment: Task 22 AUC 0.830 should not be described as a high-ranking task.*

**Response:** Corrected. Section 4.2 now identifies Tasks 23, 17, and 19 as the three strongest task-wise AUCs and explicitly describes Task 22’s 0.830 value as moderate. Table 3 retains the exact result.

**Manuscript location:** *Results 4.2; Table 3*

## R4.m4

*Comment: Relabel the speculative “Clinical Significance” column in the SHAP table.*

**Response:** Corrected. The column is now “Exploratory interpretation,” and the caption states that the entries are explanatory associations rather than validated biomarkers. Mechanistic claims such as executive planning difficulty were removed or reframed as task demands that cannot be inferred causally from SHAP.

**Manuscript location:** *Results 4.4; Table 6*

## R4.m5

*Comment: Cite Pudil et al. (1994) and a source addressing demographic confounds in handwriting assessment.*

**Response:** Completed. Pudil et al. is now Reference 39 and is used to distinguish classical floating search from GFS-SR. References 41 and 42 address cognitive impairment, education-adjusted assessment, normal ageing, and handwriting performance. They support the new confounding limitation and softened interpretation.

**Manuscript location:** *Methods 3.5; Discussion; Refs 39, 41, 42*

# Closing statement

We believe the revision now separates exploratory search scores from held-out evidence, states the methodological contribution accurately, and makes every limitation needed for responsible interpretation clear. We thank the Academic Editor and Reviewers for prompting these substantive improvements.

Sincerely,

**Tehreem Fatima**

First author, on behalf of all authors
