# Supplementary material for: Handwriting-based Alzheimer’s disease classification on the DARWIN dataset: Repeated nested validation, task-level reduction, and cross-validated explainability
Source: PLoS One. 2026 Oct 26;21(10):e0361117. doi: 10.1371/journal.pone.0361117 (PMC13704832; doi:10.1371/journal.pone.0361117)
Supplement: Supplementary file 3 [file pone.0361117.s005.docx]

Response to Reviewers

Manuscript PONE-D-26-28296R1

Handwriting-based Alzheimer’s disease classification on the DARWIN dataset: repeated nested validation, task-level reduction, and cross-validated explainability

Dear Dr. Coelho and Reviewer #4,

Thank you for assessing the revised manuscript and for identifying the remaining citation issue. We have removed the unsupported education claim and its citation from the Discussion. The analyses, results, tables, figures, reference list, and financial disclosure have not changed.

**Reviewer #4 Remaining comment**

Please verify that the citation supporting the statement regarding the influence of education on handwriting exposure/cognitive screening directly supports that claim, or provide a more appropriate reference.

**Response**

We agree. The cited handwriting study [41] does not establish that education affects handwriting exposure. We have removed the entire claim about education influencing handwriting exposure and cognitive-screening interpretation, together with its citation at that location. The paragraph now makes no claim about education's effect on handwriting. It retains the directly relevant limitation that the DARWIN analytic file has no participant-level age or education variables, so we could not compare groups or adjust the models for these characteristics. Reference [41] remains cited elsewhere for its handwriting findings; the reference list itself is unchanged.

**Manuscript change**

Section 5, Discussion, paragraph beginning ‘Demographic confounding remains unresolved’: ‘Normal ageing itself affects handwriting speed and fluency [42]. Because DARWIN’s released analytic file contains no age or education variables, we could neither compare groups nor adjust the models.’ The following sentences retain the limitation on interpreting temporal features and the recommendation for future adjusted analyses.

Sincerely,
Tehreem Fatima
On behalf of all authors
