# Supplementary material for: Handwriting-based Alzheimer’s disease classification on the DARWIN dataset: Repeated nested validation, task-level reduction, and cross-validated explainability
Source: PLoS One. 2026 Oct 26;21(10):e0361117. doi: 10.1371/journal.pone.0361117 (PMC13704832; doi:10.1371/journal.pone.0361117)
Supplement: Peer review history

# Peer review: Decision Letter 0

**DOI:** 10.1371/journal.pone.0361117.r001

**Authors:** Mohammad Shokouhifar

12 Jul 2026

Dear Dr. Fatima,

Thank you for submitting your manuscript to PLOS ONE. After careful consideration, we feel that it has merit but does not fully meet PLOS ONE’s publication criteria as it currently stands. Therefore, we invite you to submit a revised version of the manuscript that addresses the points raised during the review process.

Please submit your revised manuscript by Sep 10 2026 11:59PM. If you will need more time than this to complete your revisions, please reply to this message or contact the journal office at plosone@plos.org. When you're ready to submit your revision, log on to https://www.editorialmanager.com/pone/ and select the 'Submissions Needing Revision' folder to locate your manuscript file.

- A letter that responds to each point raised by the academic editor and reviewer(s). You should upload this letter as a separate file labeled 'Response to Reviewers'.
- A marked-up copy of your manuscript that highlights changes made to the original version. You should upload this as a separate file labeled 'Revised Manuscript with Track Changes'.
- An unmarked version of your revised paper without tracked changes. You should upload this as a separate file labeled 'Manuscript'.

If you would like to make changes to your financial disclosure, please include your updated statement in your cover letter. Guidelines for resubmitting your figure files are available below the reviewer comments at the end of this letter.

If applicable, we recommend that you deposit your laboratory protocols in protocols.io to enhance the reproducibility of your results. Protocols.io assigns your protocol its own identifier (DOI) so that it can be cited independently in the future. For instructions see: https://journals.plos.org/plosone/s/submission-guidelines#loc-laboratory-protocols. Additionally, PLOS ONE offers an option for publishing peer-reviewed Lab Protocol articles, which describe protocols hosted on protocols.io. Read more information on sharing protocols at https://plos.org/protocols?utm_medium=editorial-email&utm_source=authorletters&utm_campaign=protocols.

As the corresponding author, your ORCID iD is verified in the submission system and will appear in the published article. PLOS supports the use of ORCID, and we encourage all coauthors to register for an ORCID iD and use it as well. Please encourage your coauthors to verify their ORCID iD within the submission system before final acceptance, as unverified ORCID iDs will not appear in the published article. *Only*  the individual author can complete the verification step; PLOS staff *cannot*  verify ORCID iDs on behalf of authors.

We look forward to receiving your revised manuscript.

Kind regards,

Mohammad Shokouhifar

Academic Editor

PLOS One

**Journal Requirements:**

When submitting your revision, we need you to address these additional requirements.

1. Please ensure that your manuscript meets PLOS ONE's style requirements, including those for file naming. The PLOS ONE style templates can be found at

https://journals.plos.org/plosone/s/file?id=wjVg/PLOSOne_formatting_sample_main_body.pdf and

https://journals.plos.org/plosone/s/file?id=ba62/PLOSOne_formatting_sample_title_authors_affiliations.pdf

2. Please note that your Data Availability Statement is currently missing the DOI/accession number of each dataset OR a direct link to access each database. If your manuscript is accepted for publication, you will be asked to provide these details on a very short timeline. We therefore suggest that you provide this information now, though we will not hold up the peer review process if you are unable.

3. Your ethics statement should only appear in the Methods section of your manuscript. If your ethics statement is written in any section besides the Methods, please move it to the Methods section and delete it from any other section. Please ensure that your ethics statement is included in your manuscript, as the ethics statement entered into the online submission form will not be published alongside your manuscript.

4. Please ensure that you refer to Figures 1 to 8 in your text as, if accepted, production will need this reference to link the reader to the figure.

5. We note you have included a table to which you do not refer in the text of your manuscript. Please ensure that you refer to Tables 3 and 4 in your text; if accepted, production will need this reference to link the reader to the Table.

6. If the reviewer comments include a recommendation to cite specific previously published works, please review and evaluate these publications to determine whether they are relevant and should be cited. There is no requirement to cite these works unless the editor has indicated otherwise.

[Note: HTML markup is below. Please do not edit.]

Reviewers' comments:

Reviewer's Responses to Questions

**Comments to the Author**

1. Is the manuscript technically sound, and do the data support the conclusions?

Reviewer #1: No

Reviewer #2: Partly

Reviewer #3: Yes

Reviewer #4: Partly

**********

2. Has the statistical analysis been performed appropriately and rigorously?

Reviewer #1: I Don't Know

Reviewer #2: No

Reviewer #3: Yes

Reviewer #4: Yes

**********

3. Have the authors made all data underlying the findings in their manuscript fully available?

The PLOS Data policy

Reviewer #1: Yes

Reviewer #2: Yes

Reviewer #3: Yes

Reviewer #4: Yes

**********

4. Is the manuscript presented in an intelligible fashion and written in standard English?

Reviewer #1: Yes

Reviewer #2: Yes

Reviewer #3: Yes

Reviewer #4: Yes

**********

Reviewer #1: 1. Given that the DARWIN dataset, ML models like Random Forest and XGBoost, SFFS and SHAP methods already exist, where exactly is the main innovation of your paper? Do you consider innovation algorithmic or more in combining methods and designing a usable clinical framework?

2. Previous studies on DARWIN have also investigated different models, ensembles, feature selection and explainability. What exactly does your paper add that those studies didn't?

3. One of your contributions is the comparison of 8 models under a joint nested cross-validation. Do you consider this an independent scientific innovation or more of a standard and reproducible baseline for a fairer comparison?

3.1 Since SFFS is a well-known method for feature/task selection, what is your innovation in using SFFS? Did you just apply it to the DARWIN tasks, or was there a specific change in the way the tasks were selected? I don't see any innovation in your work to publish the paper.

4. Considering that the preprocessing includes imputation, ANOVA feature selection, standardization and calibration, are all these steps completely done inside each fold of nested cross-validation?

5. In the SFFS section, the same dataset of 174 people has been used for both task selection and performance reporting. Don't you think the higher AUC of the 7-task subset compared to the full-task baseline could be due to overfitting to the same dataset?

6. If you want to really propose the 7-task protocol as a screening protocol, wouldn't it be better to perform the task selection inside a separate outer nested validation so that the final performance is less optimistic?

7. For SFFS, light random forest with limited depth is used. Are the selected tasks stable for other models such as SVM, XGBoost or Logistic Regression, or is this subset optimized only for the same Random Forest?

8. For the application of Alzheimer's screening, false negative has a high cost. Why did you choose ROC-AUC as the main metric, not sensitivity at a fixed specificity?

9. How much is the final classification threshold set?

10. SHAP has been performed on 90 selected ANOVA features. Could clinically important features have been removed by ANOVA before SHAP?

Reviewer #2: 1. What is the configured value for the final classification threshold?

2. The principal idea behind this study has not been adequately conveyed. please explain your idea and innovation?

3. What is the difference between the idea presented in this paper and those reviewed in the existing literature?

4. Has the SFFS feature selection algorithm been optimized? If not, please explain the proposed development or extension in detail.?

Reviewer #3: The statistical comparison is limited to the Random Forest and Stacking Ensemble

models. A comprehensive statistical comparison among all classifiers using appropriate

non-parametric would provide stronger evidence for the reported performance

differences.

2. The Sequential Forward Floating Selection (SFFS) procedure and its evaluation were

performed on the same dataset. This may introduce optimistic bias in the reported

performance of the reduced seven-task protocol. The authors should discuss this

limitation more explicitly and recommend external validation of the selected task

subset.

3. Table 6 compares the proposed approach with previous DARWIN-based studies;

however, the comparison is difficult to interpret because different validation strategies

were used (80/20 split, repeated cross-validation, nested cross-validation, task-specific

evaluation, etc.). Consider adding a separate column indicating the evaluation protocol

and explicitly state that direct numerical comparisons should be interpreted cautiously.

4. Provide additional details regarding the hyperparameter search space and optimization

procedure for each machine learning model.

5. Include information about the computational resources, training time, and inference

time for the evaluated classifiers.

Reviewer #4: This manuscript presents a comparative machine learning framework for Alzheimer's disease screening using handwriting features from the DARWIN dataset, combined with a task-selection procedure and multi-level SHAP explainability analysis. The paper is well-organized, addresses a genuine gap in the DARWIN literature, and is commendably transparent about several limitations (small sample size, non-independent validation of the reduced task protocol, non-significant ensemble gain). However, several methodological issues should be resolved before the core claims can be considered fully robust and reproducible.

Major concerns:

Single-run nested cross-validation. With an outer fold size of ~35 subjects (174/5), the reported metrics show high variance (e.g., Random Forest specificity 0.835 ± 0.128). A single 5×3 nested CV run is insufficient to establish a stable performance estimate at this sample size. I recommend repeating the nested CV across multiple random seeds (e.g., 10–30 repetitions) and reporting the distribution of outer-fold AUCs rather than a single-run point estimate.

Possible leakage in the SHAP explanatory feature-selection stage. The main classification pipeline correctly performs ANOVA feature selection within training folds only. However, the 90 features used for SHAP analysis appear to be selected once, likely on the full 174-subject labelled dataset, rather than per-fold. This does not bias the Table 1 classification results, but it may inflate confidence in which features are portrayed as "dominant" in Figures 5–8, since whole-sample ANOVA selection preferentially retains features correlated with outcome in this specific cohort. Please either perform the SHAP-stage feature selection within a cross-validated wrapper and aggregate importance across folds, or explicitly state this limitation.

No reporting or control of demographic confounders. Age and education level are well-established, strong determinants of handwriting speed, pressure, and fluency independent of AD status. The manuscript does not report whether the 89 AD and 85 HC subjects are balanced on these variables, nor does it test whether the "temporal slowing" SHAP signal might partly reflect age/education differences rather than AD-specific cognitive-motor change. I recommend reporting demographic distributions by group and, if possible, a sensitivity analysis testing the correlation of top SHAP features with age/education. Absent this, statements such as "temporal handwriting features... are central contributors to AD classification" (Section 4.4/Discussion) should be softened to "associated with class membership" rather than implying a specific cognitive mechanism.

Imprecise use of "SFFS" terminology. The described task-selection procedure (forward growth followed by swap-based local refinement at fixed k) differs from classical Sequential Forward Floating Selection (Pudil et al., 1994), which interleaves forward inclusion with conditional backward exclusion steps throughout the search. I recommend either renaming the procedure (e.g., "greedy forward selection with swap-based refinement") or providing an algorithm box/pseudocode that precisely specifies the floating/backward step, termination criteria, and tie-breaking rules, both for terminological accuracy and reproducibility.

Ambiguity in ANOVA k selection for the main pipeline. Section 3.2/3.4 states that k ∈ {30, 60, 90} was "considered for the overall evaluation," but it is not clear whether k is tuned inside the inner CV loop (legitimate nested selection) or whether all three values were tried and the best reported post hoc across outer folds (a potential source of leakage/optimism). Please clarify this explicitly, as it is central to the validity of the headline Table 1 results.

Additional moderate/minor points:

Only one pairwise statistical comparison is reported (Stacking vs. Random Forest, Wilcoxon on 5 folds), despite eight classifiers being implicitly ranked in Table 1. Consider a broader comparison (e.g., Friedman test with post-hoc correction) or temper ranking language for models with overlapping confidence intervals (RF, MLP, Stacking, XGBoost, GNB all cluster around AUC 0.90–0.94).

With 450 engineered features derived from 25 tasks × 18 metrics (including features that are linear combinations of one another, e.g., total_time = air_time + paper_time), multicollinearity is likely and should be acknowledged as a caveat on SHAP importance stability, since correlated features can arbitrarily share importance across tree splits.

Table 2 (task-wise AUCs) lists Task 22 at AUC = 0.830, yet the text (Section 4.2) describes it as among "other high-ranking tasks," which is a modest overstatement relative to higher-ranked tasks (e.g., Task 17 = 0.864, Task 9 = 0.844). Consider revising this sentence for consistency with Table 2.

The "Clinical Significance" column in Table 4 offers fairly speculative mechanistic interpretations (e.g., "may reflect executive planning difficulty") for SHAP rankings; consider labeling this column explicitly as exploratory/proposed interpretation to avoid overstating what a SHAP value can support.

Consider citing Pudil, Novovičová & Kittler (1994) for the original SFFS algorithm, given the paper's explicit use of "SFFS-style" terminology, and consider adding a reference addressing demographic confounds in handwriting-based cognitive assessment.

**********

what does this mean?). If published, this will include your full peer review and any attached files.

If you choose “no”, your identity will remain anonymous but your review may still be made public.

**Do you want your identity to be public for this peer review?**  For information about this choice, including consent withdrawal, please see our Privacy Policy

Reviewer #1: No

Reviewer #2: No

Reviewer #3: No

Reviewer #4: **Yes:**  Mehrafarin Shetabi

**********

[NOTE: If reviewer comments were submitted as an attachment file, they will be attached to this email and accessible via the submission site. Please log into your account, locate the manuscript record, and check for the action link "View Attachments". If this link does not appear, there are no attachment files.]

To ensure your figures meet our technical requirements, please review our figure guidelines: https://journals.plos.org/plosone/s/figures

You may also use PLOS’s free figure tool, NAAS, to help you prepare publication quality figures: https://journals.plos.org/plosone/s/figures#loc-tools-for-figure-preparation.

NAAS will assess whether your figures meet our technical requirements by comparing each figure against our figure specifications.

# Author comment: Author response to Decision Letter 1

**DOI:** 10.1371/journal.pone.0361117.r002

24 Jul 2026

We have addressed all editorial and reviewer comments in detail. A complete point-by-point response has been uploaded as the file “PONE-D-26-28296_Response_to_Reviewers”. The revised manuscript includes repeated nested validation, comprehensive Holm-adjusted statistical comparisons, fully nested task-selection analysis, training-derived threshold evaluation, cross-validated SHAP analysis, corrected GFS-SR terminology, computational timing, demographic and multicollinearity limitations, and all requested PLOS formatting, ethics, data-availability, figure, table, and citation corrections. Please refer to the uploaded response document for each reviewer comment, our response, and the corresponding manuscript location.

Attachment Submitted filename: PONE-D-26-28296_Response_to_Reviewers.docx

# Peer review: Decision Letter 1

**DOI:** 10.1371/journal.pone.0361117.r003

**Authors:** Paulo Jorge Simões Coelho

14 Sep 2026

Dear Dr. Fatima,

Thank you for submitting your manuscript to PLOS One. After careful consideration, we feel that it has merit but does not fully meet PLOS One’s publication criteria as it currently stands. Therefore, we invite you to submit a revised version of the manuscript that addresses the points raised during the review process.

Please submit your revised manuscript by Oct 14 2026 11:59PM. If you will need more time than this to complete your revisions, please reply to this message or contact the journal office at plosone@plos.org. When you're ready to submit your revision, log on to https://www.editorialmanager.com/pone/ and select the 'Submissions Needing Revision' folder to locate your manuscript file.

- A letter that responds to each point raised by the academic editor and reviewer(s). You should upload this letter as a separate file labeled 'Response to Reviewers'.
- A marked-up copy of your manuscript that highlights changes made to the original version. You should upload this as a separate file labeled 'Revised Manuscript with Track Changes'.
- An unmarked version of your revised paper without tracked changes. You should upload this as a separate file labeled 'Manuscript'.

If you would like to make changes to your financial disclosure, please include your updated statement in your cover letter. Guidelines for resubmitting your figure files are available below the reviewer comments at the end of this letter.

If applicable, we recommend that you deposit your laboratory protocols in protocols.io to enhance the reproducibility of your results. Protocols.io assigns your protocol its own identifier (DOI) so that it can be cited independently in the future. For instructions see: https://journals.plos.org/plosone/s/submission-guidelines#loc-laboratory-protocols. Additionally, PLOS One offers an option for publishing peer-reviewed Lab Protocol articles, which describe protocols hosted on protocols.io. Read more information on sharing protocols at https://plos.org/protocols?utm_medium=editorial-email&utm_source=authorletters&utm_campaign=protocols.

As the corresponding author, your ORCID iD is verified in the submission system and will appear in the published article. PLOS supports the use of ORCID, and we encourage all coauthors to register for an ORCID iD and use it as well. Please encourage your coauthors to verify their ORCID iD within the submission system before final acceptance, as unverified ORCID iDs will not appear in the published article. *Only*  the individual author can complete the verification step; PLOS staff *cannot*  verify ORCID iDs on behalf of authors.

We look forward to receiving your revised manuscript.

Kind regards,

Paulo Jorge Simões Coelho

Academic Editor

PLOS One

Journal Requirements:

If the reviewer comments include a recommendation to cite specific previously published works, please review and evaluate these publications to determine whether they are relevant and should be cited. There is no requirement to cite these works unless the editor has indicated otherwise.

Please review your reference list to ensure that it is complete and correct. If you have cited papers that have been retracted, please include the rationale for doing so in the manuscript text, or remove these references and replace them with relevant current references. Any changes to the reference list should be mentioned in the rebuttal letter that accompanies your revised manuscript. If you need to cite a retracted article, indicate the article’s retracted status in the References list and also include a citation and full reference for the retraction notice.

[Note: HTML markup is below. Please do not edit.]

Reviewers' comments:

Reviewer's Responses to Questions

**Comments to the Author**

Reviewer #1: (No Response)

Reviewer #4: All comments have been addressed

**********

2. Is the manuscript technically sound, and do the data support the conclusions?

Reviewer #1: (No Response)

Reviewer #4: Yes

**********

3. Has the statistical analysis been performed appropriately and rigorously?

Reviewer #1: (No Response)

Reviewer #4: Yes

**********

4. Have the authors made all data underlying the findings in their manuscript fully available?

The PLOS Data policy

Reviewer #1: (No Response)

Reviewer #4: Yes

**********

5. Is the manuscript presented in an intelligible fashion and written in standard English?

Reviewer #1: (No Response)

Reviewer #4: Yes

**********

Reviewer #1: (No Response)

Reviewer #4: The authors have responded thoroughly to my previous concerns. In particular, they have implemented repeated nested cross-validation, removed the potential SHAP feature-selection leakage, clarified the nested selection of ANOVA k, corrected the SFFS terminology, expanded the statistical comparison across classifiers, acknowledged demographic and multicollinearity limitations, and moderated the interpretation of task-level and SHAP findings. The revised manuscript is substantially stronger, and my major methodological concerns have been resolved. I have only one minor point: please verify that the citation supporting the statement regarding the influence of education on handwriting exposure/cognitive screening directly supports that claim, or provide a more appropriate reference.

**********

what does this mean?). If published, this will include your full peer review and any attached files.

If you choose “no”, your identity will remain anonymous but your review may still be made public.

**Do you want your identity to be public for this peer review?**  For information about this choice, including consent withdrawal, please see our Privacy Policy

Reviewer #1: No

Reviewer #4: **Yes:**  Mehrafarin Shetabi

**********

[NOTE: If reviewer comments were submitted as an attachment file, they will be attached to this email and accessible via the submission site. Please log into your account, locate the manuscript record, and check for the action link "View Attachments". If this link does not appear, there are no attachment files.]

To ensure your figures meet our technical requirements, please review our figure guidelines: https://journals.plos.org/plosone/s/figures

You may also use PLOS’s free figure tool, NAAS, to help you prepare publication quality figures: https://journals.plos.org/plosone/s/figures#loc-tools-for-figure-preparation.

NAAS will assess whether your figures meet our technical requirements by comparing each figure against our figure specifications.

# Author comment: Author response to Decision Letter 2

**DOI:** 10.1371/journal.pone.0361117.r004

25 Sep 2026

We thank Reviewer #4 for pointing out that the citation did not directly support our statement about education and handwriting exposure. We agree and have removed the unsupported statement and its citation from Section 5 of the Discussion. The paragraph now limits its conclusion to what the DARWIN data allow: because participant-level age and education are unavailable, we could not compare the groups or adjust the models for these characteristics. We have detailed this change in the uploaded Response to Reviewers.

Attachment Submitted filename: PONE-D-26-28296_R2_Response_to_Reviewers.docx

# Editor report: Decision Letter 2

**DOI:** 10.1371/journal.pone.0361117.r005

**Authors:** Paulo Jorge Simões Coelho

7 Oct 2026

Handwriting-based Alzheimer’s disease classification on the DARWIN dataset: repeated nested validation, task-level reduction, and cross-validated explainability

PONE-D-26-28296R2

Dear Dr. Fatima,

We’re pleased to inform you that your manuscript has been judged scientifically suitable for publication and will be formally accepted for publication once it meets all outstanding technical requirements.

Within one week, you’ll receive an e-mail detailing the required amendments. When these have been addressed, you’ll receive a formal acceptance letter and your manuscript will be scheduled for publication.

An invoice will be generated when your article is formally accepted. Please note, if your institution has a publishing partnership with PLOS and your article meets the relevant criteria, all or part of your publication costs will be covered. Please make sure your user information is up-to-date by logging into Editorial Manager at Editorial Manager® and clicking the ‘Update My Information' link at the top of the page. For questions related to billing, please contact billing support.

If your institution or institutions have a press office, please notify them about your upcoming paper to help maximize its impact. If they’ll be preparing press materials, please inform our press team as soon as possible -- no later than 48 hours after receiving the formal acceptance. Your manuscript will remain under strict press embargo until 2 pm Eastern Time on the date of publication. For more information, please contact onepress@plos.org.

Kind regards,

Paulo Jorge Simões Coelho

Academic Editor

PLOS One

Additional Editor Comments (optional):

Reviewers' comments:

# Editor report: Acceptance letter

**DOI:** 10.1371/journal.pone.0361117.r006

**Authors:** Paulo Jorge Simões Coelho

PONE-D-26-28296R2

PLOS One

Dear Dr. Fatima,

I'm pleased to inform you that your manuscript has been deemed suitable for publication in PLOS One. Congratulations! Your manuscript is now being handed over to our production team.

At this stage, our production department will prepare your paper for publication. This includes ensuring the following:

* All references, tables, and figures are properly cited

* All relevant supporting information is included in the manuscript submission,

* There are no issues that prevent the paper from being properly typeset

You will receive further instructions from the production team, including instructions on how to review your proof when it is ready. Please keep in mind that we are working through a large volume of accepted articles, so please give us a few days to review your paper and let you know the next and final steps.

Lastly, if your institution or institutions have a press office, please let them know about your upcoming paper now to help maximize its impact. If they'll be preparing press materials, please inform our press team within the next 48 hours. Your manuscript will remain under strict press embargo until 2 pm Eastern Time on the date of publication. For more information, please contact onepress@plos.org.

You will receive an invoice from PLOS for your publication fee after your manuscript has reached the completed accept phase. If you receive an email requesting payment before acceptance or for any other service, this may be a phishing scheme. Learn how to identify phishing emails and protect your accounts at https://explore.plos.org/phishing.

If we can help with anything else, please email us at customercare@plos.org.

Thank you for submitting your work to PLOS One and supporting open access.

Kind regards,

PLOS One Editorial Office Staff

on behalf of

Dr. Paulo Jorge Simões Coelho

Academic Editor

PLOS One
